# Unprecedented dipole alignment in α-phase nylon-11 nanowires for high-performance energy-harvesting applications

**DOI:** 10.1126/sciadv.aay5065

**Published:** 2020-06-10

**Authors:** Yeon Sik Choi, Sung Kyun Kim, Michael Smith, Findlay Williams, Mary E. Vickers, James A. Elliott, Sohini Kar-Narayan

**Affiliations:** Department of Materials Science and Metallurgy, University of Cambridge, 27 Charles Babbage Road, Cambridge CB3 0FS, UK.

## Abstract

Dipole alignment in ferroelectric polymers is routinely exploited for applications in charge-based applications. Here, we present the first experimental realization of ideally ordered dipole alignment in α-phase nylon-11 nanowires. This is an unprecedented discovery as dipole alignment is typically only ever achieved in ferroelectric polymers using an applied electric field, whereas here, we achieve dipole alignment in as-fabricated nanowires of ‘non-ferroelectric’ α-phase nylon-11, an overlooked polymorph of nylon proposed 30 years ago but never practically realized. We show that the strong hydrogen bonding in α-phase nylon-11 serves to enhance the molecular ordering, resulting in exceptional intensity and thermal stability of surface potential. This discovery has profound implications for the field of triboelectric energy harvesting, as the presence of an enhanced surface potential leads to higher mechanical energy harvesting performance. Our approach therefore paves the way towards achieving robust, high-performance mechanical energy harvesters based on this unusual ordered phase of nylon-11.

## Teaser

α-Phase nylon-11 nanowires exhibit unprecedented dipole alignment, leading to high-performance triboelectric generators.

## INTRODUCTION

Remanent polarization in a ferroelectric polymer is the polarization that persists when an applied electric field is reduced to zero. Typically, ferroelectric polymer materials, such as poly(vinylidene fluoride) (PVDF) and its copolymers, exhibit nonzero remanent polarization following electrical poling (*1*, *2*). These materials have found use in various applications, including sensors, materials for tissue regeneration, and energy-harvesting devices (*3*–*6*). In particular, ferroelectric materials have received substantial interest for mechanical vibration-based energy-harvesting applications, such as in triboelectric generators, as the amount of accumulated charges (i.e., triboelectric charge) on the contact surface can be improved by increasing the intensity of the remanent polarization in the material (*7*–*11*). However, fabrication of polymers with strong and thermally stable remanent polarization, as required for next-generation high-performance energy harvesters, has been a long-standing issue (*12*–*14*).

In ferroelectric polymers, remanent polarization can be generated via molecular alignment (i.e., preferential crystal orientation) and resulting dipole alignment by electrical poling, (i.e., the application of an electric field higher than the coercive field of the material). The remanent polarization (*P*_r_, μC cm^─2^) obtained in this way is always much lower than the saturation polarization, as the forcibly oriented polymer molecules relax back into an equilibrium conformation once the electric field is removed. Furthermore, above the Curie temperature (*T*_C_), ferroelectric polymers readily lose their *P*_r_ since the structural phase transition occurs. These phenomena indicate that the low intensity and limited thermal stability of remanent polarization can be attributed to the molecular structure of the ferroelectric polymer. Polymers with hydrogen bonds can potentially overcome such limitations, and odd-numbered nylons, especially nylon-11, are well-known ferroelectric polymers with hydrogen bonds. As a result, through extensive hydrogen bonding, nylon-11 can exhibit better packing and a more stable molecular configuration. (It must be noted that even with the hydrogen bonds, the *P*_r_ of nylon-11 cannot exceed that of fluoropolymers because of its different constituents and molecular structures.) Furthermore, nylon-11 shows good thermal stability and ferroelectric properties, which are comparable to those of PVDF and their copolymers (*15*–*18*). However, among the various crystal structures of nylon-11, ferroelectric properties, including remanent polarization, have only been achieved in the metastable δ′-phase, with relatively sparse chain packing and random hydrogen bonds, as the only way to achieve polarization has been through electrical poling (a detailed explanation of the crystal structure of nylon-11 is provided in note S1) (*14*–*16*). In the case of the thermodynamically stable α-phase, despite its outstanding thermal stability based on denser molecular packing and well-ordered hydrogen bonds, achieving dipole alignment via electric poling has not been possible (*19*, *20*). This is because tightly packed hydrogen bonds in the α-phase restrain the rotation of dipoles up to the point of electrical breakdown, which is why the α-phase has been known as a “polar” but “nonferroelectric” phase (*21*, *22*).

Here, we have found exceptionally ordered and thermally stable dipole alignment in α-phase nylon-11 nanowires. Through a nanoconfinement effect, namely, “thermally assisted nanotemplate infiltration (TANI) method,” α-phase nylon-11 nanowires with definitive dipolar alignment have been achieved spontaneously without the need for an external electric poling field. The ideal *P*_r_ value of perfectly aligned α-phase nylon-11 was confirmed through molecular simulations. To demonstrate the formation of preferential crystal orientation in α-phase nanowires, we performed detailed x-ray diffraction (XRD) analysis using nanowires with and without the supporting nanotemplate. The remarkably high surface potential of the α-phase nanowires, corresponding to unidirectional dipole alignment, was measured directly by Kelvin probe force microscopy (KPFM), indicating that α-phase with fully aligned dipoles would have much greater net polarization than that of electrically poled ferroelectric δ′-phase. The robust thermal stability of the dipole alignment in α-phase nylon-11 nanowires was also confirmed through studies of surface potential and molecular structure changes before and after thermal annealing. Correspondingly, a triboelectric energy generator based on α-phase nylon-11 nanowires fabricated via the TANI method showed 34 times higher output power density as compared to an aluminum-based device, when subjected to identical mechanical excitations.

## RESULTS

### Intensity of ideal polarization

**Fig. 1:**
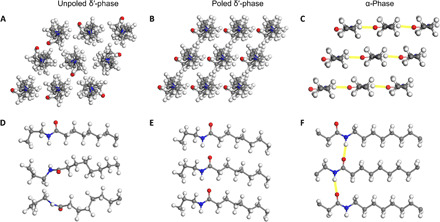
Molecular structure and chain packing of nylon-11. (**A** and **D**) Schematics show packing of nylon-11 monomeric units in unpoled δ′-phase nylon-11 viewed along the *c* axis and *b* axis, respectively. All molecules lie in a random orientation. (**B** and **E**) The crystal structures and molecular packing of the poled δ′-phase. All the oxygen atoms point to the same direction. (**C** and **F**) Crystal structure of α-phase nylon-11. The fully stretched molecules in trans configuration with sheets of strong hydrogen bonding gives the α-phase a relatively well-ordered crystal structure. The yellow lines indicate intermolecular hydrogen bonding. Gray spheres, carbon atoms; red spheres, oxygen atoms; blue spheres, nitrogen atoms; white spheres, hydrogen atoms.

As a semicrystalline polymer, nylon-11 has at least three crystal structures referred to as triclinic (α and α′), monoclinic (β), and pseudo-hexagonal (γ, δ, and δ′) (*2*, *23*). Among them, only the metastable pseudo-hexagonal phases, such as the δ′-phase, display ferroelectric properties due to sparse chain packing and random hydrogen bonding. As Fig. 1 (A and D) illustrates, the unpoled δ′-phase has randomly oriented nylon-11 chains within a pseudo-hexagonal unit cell, resulting in the cancellation of dipole moments (*23*). Additional mechanical drawing and subsequent electrical poling allow chains to rotate such that the amide groups point in the same direction, resulting in a net dipole moment (Fig. 1, B and E) (*14*–*16*). In contrast, the α-phase can adopt a well-aligned molecular structure in the triclinic unit cell without stretching and/or high-voltage poling (Fig. 1, C and F). This is because the hydrogen bonds are organized into well-defined sheets held together by van der Waals interactions, with the amide groups of adjacent chains located at about the same height along the chain axis (*23*). As a result, the dipole moment perpendicular to the chain axis points along a single direction. However, it must be noted that such unidirectional dipole moments of α-phase are limited to a localized crystalline region, and the net-polarized directions of such crystalline regions are randomly determined during the crystallization process. Furthermore, it is also impossible to align every dipole in the α-phase structure because of the constraints on rotation of the hydrogen-bonded molecules up to the point of electrical breakdown. As a result, the net polarization of pristine α-phase (bulk) sample is much smaller than that of electrically poled δ′-phase sample.

So how high can the polarization be when the dipoles in bulk α-phase are fully aligned? To evaluate the possible maximum polarization in nylon-11, we estimate the “ideal” *P*_r_ values of perfectly aligned δ′-phase and α-phase by conducting molecular-scale simulations (*24*) (details of simulation process are discussed in note S2). To a first-order approximation, the *P*_r_ scales linearly with the dipole moment and the crystallinity (*12*, *25*). Using molecular simulation, the dipole moment for individual molecules can be measured by assigning partial atomic charges to the atoms. For a minimum repeating unit, the dipole moments per unit cell of 88 × 10^─30^ and 101 × 10^─30^ C m were obtained for the δ′- and α-phase, respectively. In the case of perfectly aligned δ′-phase, assuming a crystallinity of about 40%, the calculated *P*_r_ was 3.2 μC cm^─2^; this is in good agreement with the experimentally determined value of about 5.0 μC cm^─2^ (*2*). In contrast, the α-phase with the same crystallinity showed *P*_r_ of 7.5 μC cm^─2^, meaning that α-phase with fully aligned dipoles would have much greater net polarization than that of electrically poled ferroelectric δ′-phase. This is because the fully stretched chain structure and well-aligned hydrogen bonded sheets in the α-phase maximize the dipole moment per monomer unit (*25*). In addition, considering the crystal structure, the distance between adjacent molecules in the triclinic α-phase is much smaller than that in the pseudo-hexagonal δ′-phase (*26*).

### Nanoconfinement-induced preferential crystal orientation

**Fig. 2:**
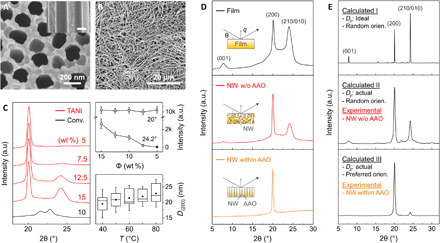
Nanowire morphology and x-ray analysis. (**A**) SEM image of the nanotemplate surface with 200 nm pores. Inset shows the template cross section. (**B**) SEM image of template-freed nanowires fabricated by the TANI method. (**C**) Left: XRD patterns of nanowires fabricated by the conventional template-wetting (black) and the TANI (red) methods. Top right: Changes in the average intensities of the XRD peak at 20° and 24.2° as a function of solution concentration. Bottom right: Changes in the crystallite sizes perpendicular to the (200) planes (*D_(200)_*) of 5 wt % samples as a function of processing temperature. ■, middle line, and top and bottom boundaries indicate mean, median, and 75 and 25% values, respectively. a.u., arbitrary units. (**D**) XRD patterns of α-phase nylon-11 film (black) and nanowires (NW) without (red) and within (orange) a nanoporous AAO template. Inset schematics display the morphology of sample and the direction of x-ray beam (θ) with a scattering vector (*q*). (**E**) XRD patterns of calculated (black) and experimentally observed (red and orange) α-phase nylon-11 nanowires. Calculated patterns I and II assumed random orientation of crystals with large (>100 nm) and small crystallite sizes (*D_200_*) (~25 nm), respectively. Calculated pattern III assumed preferred crystal orientation with actual *D_200_*.

To align the dipoles in α-phase nylon-11 nanowires, we developed the TANI method as an effective nanoconfinement technique (details of the experimental process are given in Materials and Methods and note S3) (*27*, *28*). This is because, to date, nylon-11 nanowires with thermodynamically stable α-phase have never been realized by conventional template-wetting methods because of the difficulties associated with this synthetic route (*11*, *29*). Figure 2A shows scanning electron microscope (SEM) images of an anodized aluminum oxide (AAO) nanoporous template. The top surface and cross-sectional images indicate that the pore size is around 200 nm. The morphology of the nanowires fabricated by the TANI method is displayed in Fig. 2B. Long chain-shaped nanowires with uniform width (200 nm) and length (60 μm) were detected after the AAO was dissolved using mild acid. These nanowire dimensions are similar to that of the template pore channels. The surface morphology of the nanowire was measured by atomic force microscopy (AFM). Compared to the nylon-11 film, the nanowires showed a uniform and smooth surface topography without grain boundaries (figs. S2 and S3). Note that this fabrication method can be scaled up as TANI is a nonvacuum and relatively low temperature process, and α-phase nanowires within the AAO template are relatively easy to handle (note S4).

Detailed crystal structure characterization was carried out by XRD. It has been reported that an α-phase nylon-11 film shows diffraction peaks at 2θ = 7.8°, 20°, and 24.2° (*23*). However, the nanowires fabricated by a conventional template-wetting method were found to generate diffraction patterns with weak intensity of peaks at 2θ = 21.6° and 22.8° (Fig. 2C, left, black) (*29*). This result indicates that the α-phase nylon-11 nanowires with desirable crystallinity could not be obtained through a conventional nanoconfinement method. In contrast, the nanowires fabricated by our TANI method displayed the same peak position, as the reported α-phase film, with much stronger diffraction intensities than that of conventionally generated nanowires (Fig. 2C, left, red). This means that the solvent-vapor–filled closed-heating system of the TANI method effectively mimics the slow crystallization process of typical α-phase film fabrication techniques by suppressing the speed of solvent evaporation within the nanopores. Furthermore, the TANI method allowed more precise control of the crystal structure, wherein we were able to manipulate the rate of crystallization by adjusting both the solution concentration and the processing temperature. As shown in Fig. 2C (left and right top), the relative intensity of the peak at 2θ = 24.2° gradually decreased with decreasing the solution concentration, while that of the 20° peak was maintained within the error range. This is because the more dilute solution enabled further decrease of the crystallization speed by increasing the free volume of the polymer chains. Considering that each peak corresponds to a specific lattice plane, the resulting diffraction pattern with only one distinct peak from 5 weight % (wt %) solution indicates that more aligned molecular structures could be achieved through the TANI method. Additional heating also allowed us to control the polymer crystallization process within the nanopores. The changes in the crystallite size perpendicular to (200) plane (*D_(200)_*) of 5 wt % samples as a function of processing temperature showed that the average *D_(200)_* gradually increased with processing temperature (Fig. 2C, right bottom). This indicates that the additional heating enabled an increase in the chain mobility, which is a driving force for molecular reorientation and alignment. (It must be noted that the errors in the *D_(200)_* plot are attributed to deviations between different samples. Experimental errors, originating from XRD measurement setting and profile fitting, are within a 2-nm range.) These XRD results imply that the desired α-phase nanowires could be achieved by the TANI process.

To confirm the crystallography of the nanowires, α-phase nylon-11 films were fabricated for comparison. Figure 2D shows the XRD patterns of the α-phase film (black) and template-freed nanowires fabricated by the TANI method, using 5 wt % solution and 80°C heating (red). The α-phase nylon-11 film displayed two distinct peaks at 2θ = 20° and 24.2° and one small peak at 7.8° corresponding to (200), (210/010), and (001) planes, respectively (*23*). In nanowires without the AAO template, identical peak positions with α-phase film were also observed, indicating that the TANI method enabled fabrication of α-phase nylon-11 nanowires. Notably, the diffractogram of the template-freed nanowires showed a much sharper peak for the (200) plane with a smaller full width at half maximum (FWHM) than that of α-phase film, resulting in much larger *D_(200)_* in nanowires (25 nm) than that of the film (16 nm). Furthermore, both α-phase nanowires and film samples showed a similar degree of crystallinity of ~48% (details of crystallinity calculation are discussed in note S5). Considering that most nanowires fabricated via a conventional template-wetting process showed poor crystallinity than that of films with the same crystal structures, these results suggest that the TANI method does, in fact, enable the generation of highly crystalline α-phase nanowires with larger crystal sizes compared to the α-phase film (*30*, *31*). The thermal behavior of developed nylon-11 nanowires also confirms that the TANI method synthesized the nanowires on the basis of the nanoconfinement effect (note S6). The chemical bonds in α-phase nanowires were studied using Fourier transform infrared (IR) spectroscopy measurements (note S7). In fig. S8, within the same IR spectrum, much higher relative peak intensity at both N─H stretching and amide I regions was observed from the α-phase nanowires. Considering that the N─H stretching (3300 cm^─1^) and the amide I (1635 cm^─1^) bands reflect the overall distribution of hydrogen-bonded strengths and local ordering of hydrogen bonds, respectively (*16*, *32*, *33*), it can be inferred that the TANI method further enabled well-ordered crystal growth, based on the formation of hydrogen bonds.

The direction of molecular orientation was verified by detailed XRD analysis of the nanowires within the nanoporous AAO template (Fig. 2D, orange). In principle, a single crystal sample examined in reflection mode would produce only one family of lattice planes with scattering vectors (*q*) normal to the sample surface. This means that if the nanowires have preferential crystal orientation, then the discrepancy in the diffractograms between the vertically aligned nanowires and randomly positioned nanowires would indicate the direction of crystal orientation. As discussed, the template-freed α-phase nylon-11 nanowires showed two distinct peaks at 2θ = 20° and 24.2° and one small peak at 7.8° corresponding to (200), (210/010), and (001) planes, respectively (Fig. 2D, red). In contrast, α-phase nanowires within the AAO template had only one distinct diffraction peak at 2θ_(200)_ = 20° (Fig. 2D, orange). The lack of intensity from (001) and (210/010) planes implies that the nanowires were, in fact, oriented, such that these peaks were not visible in reflection mode geometry. A rocking curve on the (200) reflection with a peak width of ~8° also confirmed this observation (note S8). These results indicate that the nanowires fabricated by the TANI method had preferential crystal orientation with the molecular chain axis perpendicular to the nanowire length direction, consistent with previous reports (*34*–*36*).

The molecular simulation results validate the determinations of crystallite size (*D_p_*) and preferential crystal orientation in α-phase nanowires (Fig. 2E). With the assumption of an ideal *D_p_* (>100 nm) and random crystal orientation (*37*), the simulated powder diffraction pattern displayed the highest peak at 2θ_(210/010)_ = 24.2° with the second highest peak at 2θ_(200)_ = 20° (Fig. 2E, top). However, introducing the experimentally measured *D*_200_ values of 25 nm changed the order of the highest peaks from 24.2° to 20° and broadened the diffraction pattern (Fig. 2E, middle). Considering that an amorphous region is likely to give rise to a broad diffraction peak at about 22.2°, the simulated data gave a good match to the experimental data from template-freed α-phase nanowires (red dot). Note, however, that the other peaks were broader, which implies that the crystallite size in these directions is smaller than that perpendicular to (200) planes. Last, applying preferential crystal orientation calculated by Rietveld-Toraya equation (*38*, *39*) resulted in a diffractogram with a remarkably high and sharp peak at 2θ_(200)_ = 20° (Fig. 2E, bottom), showing good agreement with the experimentally measured diffraction patterns of α-phase nanowires within the AAO template (orange dot). The agreement of relative peak intensity and FWHM between calculated and experimental results confirms that the relatively large crystals in the α-phase nanowires were indeed preferentially aligned and that the (200) planes were perpendicular to the axis of the nanowires.

### Surface potential of α-phase nanowires

It is believed that surface potential and resulting triboelectric charge on the surface can be improved by increasing the intensity of the net dipole moment in the material (*7*–*11*). In addition, such net dipole moment in nylon-11 results mainly from alignment of dipoles because the *P*_r_ value is close to zero when the dipole density approaches zero in oriented and poled nylon-11 (*40*, *41*). This indicates that dipole alignment generated by the preferential crystal orientation arising from the nanoconfinement effect can enhance the surface potential, and the intensity of surface potential has a close relationship with the degree of molecular orientation (*42*).

**Fig. 3:**
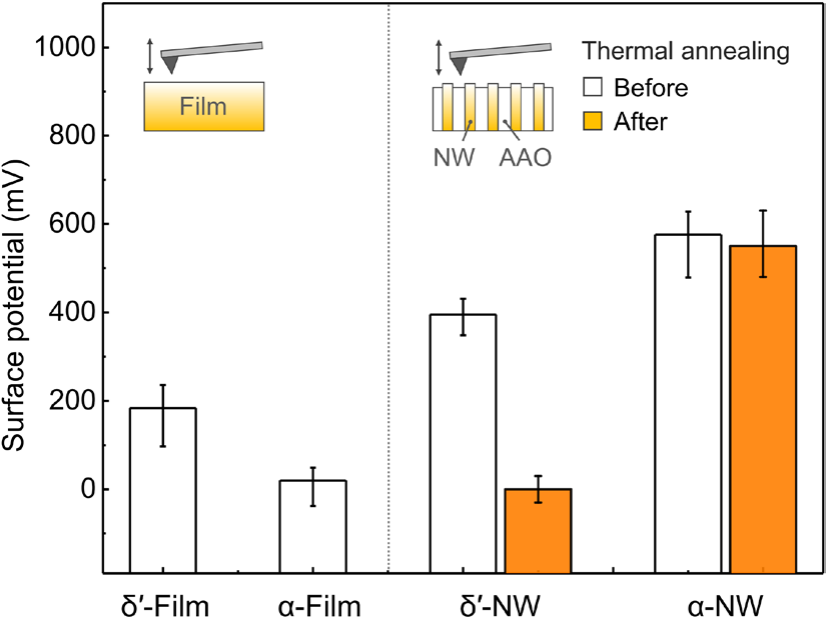
Surface potential analysis. Plots of the surface potential of various films and nanowires. Thermal stability of nanowire samples was also investigated by surface potential measurement before (white bar) and after (orange bar) thermal annealing at 165°C. Inset schematics indicate the way to measure the surface potential using KPFM.

To investigate the surface potential of α-phase nanowires, detailed analysis was conducted by KPFM (details of the KPFM technique and measurement procedure are provided in note S9). Although the *P*_r_ of ferroelectric materials has been observed by polarization–electric field (*P-E*) hysteresis loops, the net dipole moment of nonferroelectric materials cannot be measured in this way as they do not show such hysteretic behavior. In contrast, KPFM can measure the surface potential of a material, and it has been shown that net dipole moment contributes to the magnitude of the surface potential (*7*, *43*, *44*). When we compare the surface potential of nylon-11 nanowires and unpoled films, the nanowires showed much higher values than the films with the same crystal structure (Fig. 3 and fig. S11). [The top surface of nanowires within the AAO template sample is filled with nanowire tips; thus, the influence of AAO template on surface potential can be ignored (fig. S12).] Both δ′- and α-phase nanowire samples showed a 2- and 30-fold increase in surface potential compared to those of the corresponding film samples, respectively. These results indicate that the nanoconfinement effect during crystal growth effectively aligned the dipoles and generated a strong net dipole moment in the nanowires (*45*, *46*). It must be noted that the surface potential of the α-phase nanowire (576 mV) was much higher than that of δ′-phase nanowires (395 mV). This is in good agreement with our *P*_r_ calculations from molecular simulations and indicates that the TANI method gives rise to strong surface potential in α-phase nylon-11 by nanoconfinement-induced molecular ordering. (Note that when we compare the surface potential of δ′-phase and α-phase films, molecular ordering cannot be the major factor for the surface potential because both films are unpoled. Therefore, other minor factors, such as crystal size and surface roughness, should be considered to approximate the surface potential of those unpoled films.)

Thermal stability tests confirm the notable contribution of hydrogen bonding to the changes in dipolar orientation. After a thermal annealing process at 165°C, the surface potential of the δ′-phase nanowires dropped from 395 to 0 mV (Fig. 3B, orange bar). This is because the preferential crystal orientation in metastable δ′-phase disappeared because of thermal agitations (*23*). In contrast, the α-phase nanowires sustained their high surface potential (~570 mV) within the error range, even after thermal annealing at high temperature, indicating that dipole alignment in α-phase nanowires can be maintained during annealing process. The changes in XRD patterns after thermal annealing further confirmed the thermal stability of molecular configuration in α-phase nanowires (fig. S13). In the case of δ′-phase nanowires, the intensity of 21.6° peak, corresponding to the (*hk*0) planes, decreased after thermal annealing, while the intensity of peak at 2θ = 20.4° increased (fig. S13A). In contrast, the peak positions and intensities in diffractograms of α-phase nanowires before and after 165°C annealing were found to be almost the same (fig. S13B). This means that the molecular configuration within α-phase nanowires was thermally stable, and the resulting preferential crystal orientation could therefore be maintained up to near the melting temperature, as a result of the strongly hydrogen-bonded, well-ordered, and highly packed molecular structure.

**Fig. 4:**
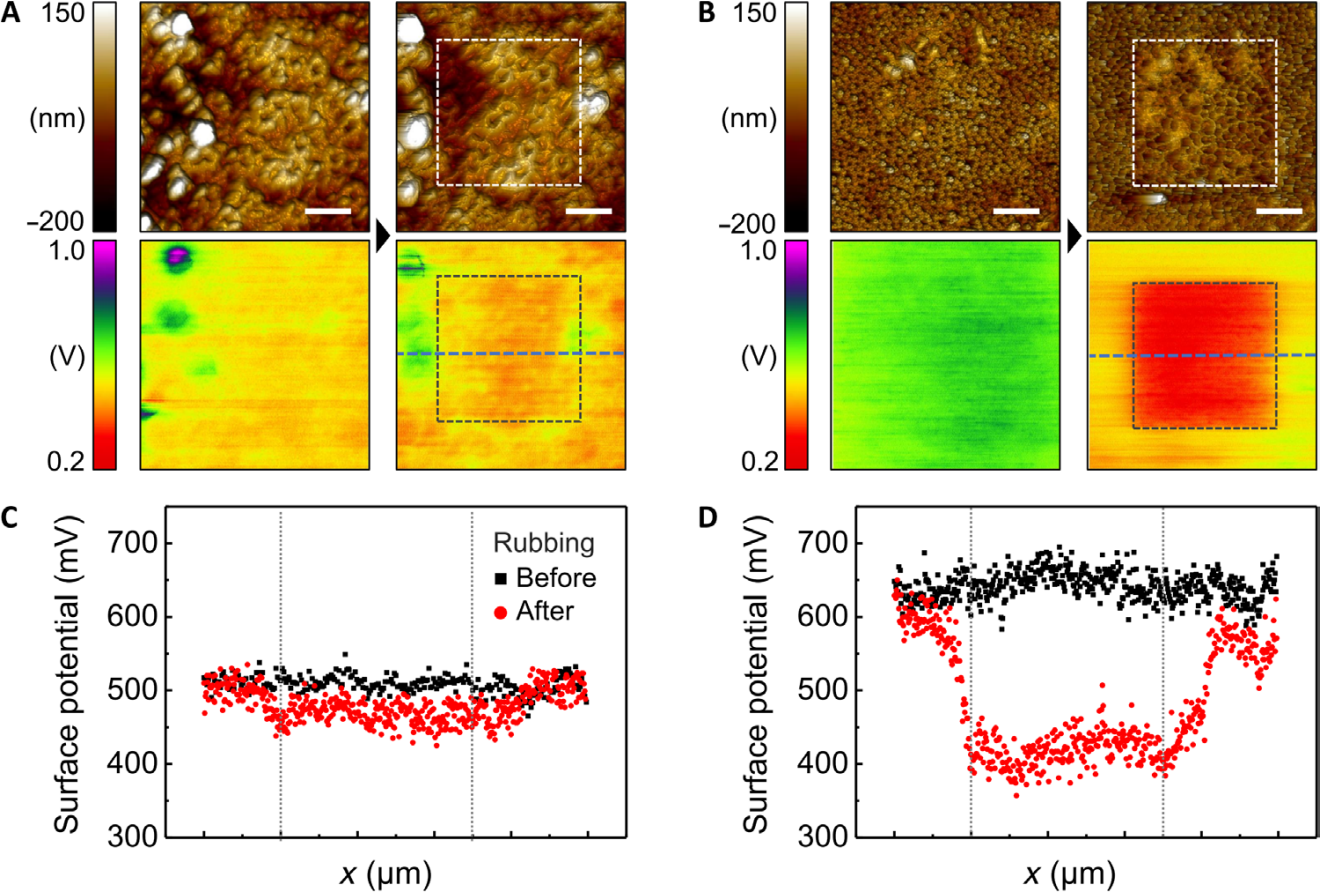
Surface potential analysis before and after rubbing process. (A and C) δ′-phase and (B and D) α-phase nanowires. (A and B) AFM topology and surface potential images before (left) and after (right) rubbing process. Dashed squares, rubbing area. Scale bars, 1 μm. Blue dashed line, the data acquisition position for plotting. (C and D) Surface potential changes before (black square) and after (red circle) rubbing process.

To verify the enhanced charge accumulation ability resulting from a higher net dipole moment, we measured the changes of surface potential before and after mechanical rubbing. This is because the accumulated charges on the surface can be transferred by contact with other materials having different work functions, and the friction of the AFM tip on the surface of the material induces such an effect (*43*). In particular, the intensity of remanent polarization in the ferroelectric polymer affects the amount of transferred charges during the rubbing process due to the direction of polarization and the degree of dipole orientation, as well as the change in charge affinity of the surface (*8*). We measured the surface potential of pristine δ′- and α-phase nanowire samples before and after mechanical rubbing (Fig. 4). After rubbing, the average surface potential of δ′-phase nanowires dropped from 510 to 469 mV, indicating that the accumulated charge on the surface of δ′-phase nanowires (corresponding to a surface potential change of 41 mV) was transferred via the AFM tip. In the case of α-phase nanowires, much larger changes in surface potential of 224 mV were obtained after rubbing (646 to 422 mV). These results indicate that α-phase nanowires maximize the charge accumulation ability because of their dipole alignment. Comparison with the α-phase film also confirms that the increased charge transfer could be attributed to the dipole alignment in the nanowires, which was otherwise absent in the film (fig. S14).

### Nanowire-based energy-harvesting devices

As a practical demonstration, we propose triboelectric generators for energy-harvesting applications. To achieve high levels of energy-harvesting performance, materials with electron-donating tendencies must be paired with those with electron-accepting tendencies, and nylon-11 belongs to the less explored family of synthetic and organic electron-donating materials (*11*). On the basis of α-phase nylon-11 nanowires fabricated by the optimized TANI method, we developed a contact-separation mode triboelectric generator with an area of 3.14 cm^2^. An Al film and δ′-phase nanowire-based devices were also prepared to compare the device performances (note S10).

**Fig. 5:**
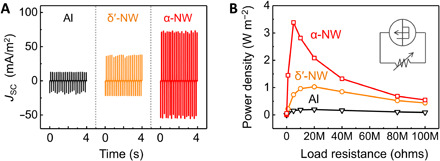
Triboelectric generator performance. (**A**) Short-circuit output current densities of triboelectric generators with different combinations of materials: Al (black), δ′-phase nanowire (orange), and α-phase nanowire (red). (**B**) The power density of the same devices as a function of load resistance. The power density is calculated by multiplying the current density squared with the load resistance.

Figure 5A shows the short-circuit current density (*J*_SC_) measured in response to the periodic impacting at a frequency of 5 Hz and amplitude of 0.7 N with 0.5 mm in an energy-harvesting setup that has been previously described (*27*). The Al-based device showed a peak *J*_SC_ of ~13 mA m^−2^. Because of the better charge-donating property of nylon and dipole alignment effect, higher device performance was observed from a δ′-phase nanowire-based triboelectric generator with *J*_SC_ of ~38 mA m^−2^ than from the Al-based device, consistent with our previous results (*11*). The α-phase nanowire-based device displayed further enhanced output performance with a peak *J*_SC_ of ~74 mA m^−2^ likely due to the much higher net dipole moment. The peak output power densities of 3.38, 1.03, and 0.099 W m^−2^ were observed from α-phase nanowires, δ′-phase nylon-11 nanowire, and Al-based device under impedance-matched conditions at a load resistance of ~5, 20, and 20 megohms, respectively (Fig. 5B and fig. S16A). The observed output power from α-phase nanowire-based triboelectric generator was ~3 times and ~34 times higher than those of δ′-phase nanowires and the Al-based device, respectively. An output power comparison between α- and δ′-phase nanowires-based devices illustrates that the much higher net dipole moment in closely packed and aligned molecular structure of α-phase nanowire contributed to the enhancement of device performance, in good agreement with the results of modeling and surface potential analysis (a detailed explanation of the triboelectric charge transfer process is provided in note S11). It must be noted that those energy-harvesting performances are originated not from piezoelectricity but from triboelectricity of nylon-11 nanowires, considering the properties of nanowires and the design of energy-harvesting setup (note S12). In terms of stability, the α-phase nylon-11 nanowire-based triboelectric generator exhibited a negligible change in output current density over the entire period of fatigue testing (≈540,000 cycles) and during long-term reliability test (~2 weeks), demonstrating the high mechanical stability of the dipole alignment in α-phase nanowires and the robustness of the nanowire-based device, respectively (figs. S16D and S17). It must be noted that the mechanical stiffness of α-phase nanowires is much higher than that of the δ′-phase nanowires because of the large crystallite with well-ordered hydrogen bonding (*47*). Although no abrasion was observed during the fatigue test in either the α- or δ′-phase nanowires, such a stiffness difference implies that the α-phase nanowire device is more appropriate for use in friction-based devices, including triboelectric energy harvesters.

## DISCUSSION

Nylon-11 nanowires exhibiting dipole alignment with an unprecedented intensity of net dipole moment and thermal stability have been fabricated. Through the nanoconfinement effect of our TANI method, α-phase nylon-11 nanowires with dipole alignment were successfully achieved. The larger crystallite size and improved alignment of hydrogen bonds were confirmed by XRD and IR measurements, while molecular simulation was used to interpret the diffraction data and to shed light on the mechanism behind the preferential crystal orientation. The intensity of net dipole moment and thermal stability of dipole alignment were also investigated by analyzing the changes in surface potential through KPFM measurements. Consequently, we have verified that, because of the ordered crystalline regions and higher molecular packing density, the net dipole moment of α-phase nylon-11 can be much higher than that of poled ferroelectric δ′-phase. Furthermore, the strong hydrogen bonding, which has previously been considered as a serious disadvantage for the polarization of nylon-11, actually serves to enhance the stability of the molecular structure, resulting in a constant net dipole moment up to near the melting temperature. When α-phase nylon-11 nanowires were incorporated in triboelectric generators, the resulting output power was observed to be ~3 times and ~34 times higher than those of δ′-phase nanowires and Al-based device, respectively. This work provides a new insight for both nanomaterial and nanofabrication methods to develop strong and thermally stable dipole alignment for next-generation high-performance energy-harvesting applications.

## MATERIALS AND METHODS

### Materials

Nylon-11 (polyamide-11), [─NH(CH_2_)_10_CO─]*_n_*, with molecular weights (*M*_w_) of 201.31 g/mol, was purchased from Sigma-Aldrich. Calculated weight percent nylon-11 solutions were prepared in formic acid (Sigma-Aldrich, reagent grade ≥95%) at 70°C.

### Fabrication of nylon-11 nanowires

To prepare the nanowires, 25-mm-diameter AAO templates (Anopore, Whatman) with a pore diameter of 200 nm and a thickness of 60 mm were placed on an 800-μl nylon-11 solution droplet. In the case of a conventional template-wetting method, the AAO template was placed on the solution and was then left at room temperature for at least 24 hours with no protective covering (*29*). As the formic acid naturally evaporated through the pores, the solution was drawn up through the pores via capillary forces, and the nylon-11 was able to crystallize into nanowires. In the case of the TANI method for the α-phase nanowires, the AAO template was attached to a square glass slide before placing on top of the solution to limit the exposure of the template’s top surface to the air, thus limiting the rate of formic acid evaporation. In addition, a lid was placed over the sample to further reduce exposure to the surrounding air and to allow the local environment to become saturated with formic acid vapor, and the sample was then placed on a hot plate. The δ′-phase nanowires were produced by maximizing the evaporation speed of the solution (*11*). The AAO template was placed on top of a drop of 17.5 wt % nylon-11 solution in accordance with the conventional template-wetting method. No additional protective layers were added, and the solution was not heated during the crystallization. To control the crystallization rate of the solution, assisted gas flow with a speed of ~3 m s^─1^ was introduced upon the AAO template using a portable mini fan placed immediately next to the floating template. The rate of assisted gas was controlled by fan rotation speed and measured by an anemometer. The whole fabrication process proceeded under room temperature.

### After treatment

For accurate characterization, the thin nylon-11 film that formed underneath the AAO template had to be removed. To do so, excess material was scraped off using a razor blade. Next, formic acid was warmed on a hot plate to 80°C and swabbed over the template bottom surface using a cotton bud. Once the thin nylon-11 films had been removed, the nanowire-filled template was washed in deionized water and dried at room temperature. To obtain the template-freed nanowires, the nanowire-filled template was immersed in a 40 volume % phosphoric acid solution for 4 hours. To achieve the assembled (and template-freed) nanowire film, acid-immersed nanowires should be lifted off by silicon wafer from the surface of acid solution. The assembled film was then washed carefully in deionized water and dried at room temperature.

### Fabrication of nylon-11 films

The α-phase nylon-11 films were produced by casting the nylon-11 solution onto the hot plate (~80°C), and a lid was placed over the sample to reduce the crystallization speed.

### Fabrication of triboelectric energy generators

To fabricate the triboelectric energy generators, a 100-nm-thick Au layer was deposited on the bottom side of the nanowires-filled AAO template by using benchtop sputter (k550 Emitech). As a counterpart material, a 100-μm-thick Teflon film was prepared, and Au was sputtered on the bottom side of the Teflon film. A 24-mm-thick aluminum film with the same diameter of 2 cm was also prepared to compare the triboelectric generator performance with the nanowire-based devices. The mechanical input was generated using a vibrational impacting system, where a permanent magnetic shaker (LDS Systems V100) was connected to an amplifier (LDS Systems PA25E-CE) driven by a signal generator (Thurlby Thandar TG1304) to generate vibration motion of the impacting arm based on a programmed signal in the signal generator. The impacting arm underwent periodic oscillations at frequency *f* (*27*). Energy-harvesting data in the form of output voltages and currents were collected by two different data acquisition modules: multimeter (Keithley 2002) for voltage and picoammeter (Keithley 6487) for current measurement. A top-down schematic of this triboelectric generator system, including the actuating and data-collecting configuration, is shown in fig. S15.

### Characterization

Three-dimensional molecular images of nylon-11 were rendered using BIOVIA Materials Studio (Dassault Systèmes BIOVIA). The morphology of the nanowires was investigated using field-emission scanning electron microscopy (FEI Nova NanoSEM) and AFM (Bruker MultiMode) with an antimony *n*-doped Si (tip radius, <35 nm; resonance frequency, 150 kHz). Detailed crystal structural characterization was carried out by an XRD machine (Bruker D8) with Cu Kα radiation (λ = 0.15418 nm). The sample was placed on a highly *p*-doped silicon substrate during the XRD measurement. The size of corresponding crystals (*D_p_*) was calculated from the diffraction peaks using Scherrer equation (*48*): *D_p_* = (K × λ)/(*B* cos θ), where *K* is the Scherrer constant, *B* is the FWHM of diffraction peak (in radians), and θ is the diffraction peak position (angle). To help optimize the process, the XRD data from more than 90 samples were measured to assess changes in *D_p_* and relative intensities of the peaks as a function of processing parameters. The degree of crystallinity (χ) was calculated by calorimetry and XRD methods (*49*). The differential scanning calorimetry (DSC) data were measured at a scanning rate of 5°C/min using TA Instruments Q2000 DSC to determine the thermal and structural properties of nylon-11 nanowires, from which the melting temperature (*T*_m_) and the melt crystallization temperature (*T*_c_) were recorded from the first heating. KPFM measurements were carried out using Bruker MultiMode 8 in the noncontact amplitude modulation (AM-KPFM) mode with 2-V AC signal, and an antimony (*n*)-doped Si tip (MESP-RC-V2, Bruker) with a nominal radius of ~35 nm, a resonant frequency of ~150 kHz, and a nominal spring constant of 5 N m^−1^ was used. The KPFM measurements were performed under the same measuring conditions for all samples (temperature = 21°C, humidity = 17%). The rubbing procedure was pursued for one time in a contact mode with a scan rate of 1 Hz, a scan area of 5 μm^2^, and a contact force of 30 nN. Film thickness was measured using a stylus surface profilometer (Veeco Dektak 6M).

## Supplementary Material

aay5065_SM.pdf

## SUPPLEMENTARY MATERIALS

Supplementary material for this article is available at http://advances.sciencemag.org/cgi/content/full/6/24/eaay5065/DC1

## References

[1] Lovinger A. J., Ferroelectric polymers. Science 220, 1115–1121 (1983). 17818472 10.1126/science.220.4602.1115

[2] H. S. Nalwa, *Ferroelectric Polymers: Chemistry: Physics, and Applications* (Marcel Dekker Inc., 1995).

[3] Horiuchi S., Tokura Y., Organic ferroelectrics. Nat. Mater. 7, 357–366 (2008). 18432209 10.1038/nmat2137

[4] Persano L., Dagdeviren C., Su Y., Zhang Y., Girardo S., Pisignano D., Huang Y., Rogers J. A., High performance piezoelectric devices based on aligned arrays of nanofibers of poly(vinylidenefluoride-co-trifluoroethylene). Nat. Commun. 4, 1633 (2013). 23535654 10.1038/ncomms2639

[5] Rajabi A. H., Jaffe M., Arinzeh T. L., Piezoelectric materials for tissue regeneration: A review. Acta Biomater. 24, 12–23 (2015). 26162587 10.1016/j.actbio.2015.07.010

[6] Valentini R. F., Vargo T. G., Gardella J. A. Jr., Aebischera P., Electrically charged polymeric substrates enhance nerve fibre outgrowth in vitro. Biomaterials 13, 183–190 (1992). 1567943 10.1016/0142-9612(92)90069-z

[7] Lee J.-H., Hinchet R., Kim T. Y., Ryu H., Seung W., Yoon H. J., Kim S. W., Control of skin potential by triboelectrification with ferroelectric polymers. Adv. Mater. 27, 5553–5558 (2015). 26292202 10.1002/adma.201502463

[8] Lee K. Y., Kim S. K., Lee J.-H., Seol D., Gupta M. K., Kim Y., Kim S.-W., Controllable charge transfer by ferroelectric polarization mediated triboelectricity. Adv. Funct. Mater. 26, 3067–3073 (2016).

[9] Seung W., Yoon H.-J., Kim T. Y., Ryu H., Kim J., Lee J.-H., Lee J. H., Kim S., Park Y. K., Park Y. J., Kim S.-W., Boosting power-generating performance of triboelectric nanogenerators via artificial control of ferroelectric polarization and dielectric properties. Adv. Energy Mater. 7, 1600988 (2016).

[10] Wang J., Wu C., Dai Y., Zhao Z., Wang A., Zhang T., Wang Z. L., Achieving ultrahigh triboelectric charge density for efficient energy harvesting. Nat. Commun. 8, 88 (2017). 28729530 10.1038/s41467-017-00131-4 PMC5519710

[11] Choi Y. S., Jing Q., Datta A., Boughey C., Kar-Narayan S., A triboelectric generator based on self-poled Nylon-11 nanowires fabricated by gas-flow assisted template wetting. Energ. Environ. Sci. 10, 2180–2189 (2017).

[12] Li M., Wondergem H. J., Spijkman M. J., Asadi K., Katsouras I., Blom P. W., de Leeuw D. M., Revisiting the δ-phase of poly(vinylidene fluoride) for solution-processed ferroelectric thin films. Nat. Mater. 12, 433–438 (2013). 23503012 10.1038/nmat3577

[13] Lee J. W., Takase Y., Newman B. A., Scheinbeim J. I., Effect of annealing on the ferroelectric behavior of nylon-11 and nylon-7. J. Polym. Sci. B Polym. Phys. 29, 279–286 (1991).

[14] Takahashi Y., Shimomura M., Kutani M., Furukawa T., Ferroelectric switching characteristics and crystal structure of nylon 11. Polym. J. 29, 234–239 (1997).

[15] Lee J. W., Takase Y., Newman B. A., Scheinbeim J. I., Ferroelectric polarization switching in nylon-11. J. Polym. Sci. B Polym. Phys. 29, 273–277 (1991).

[16] Scheinbeim J. I., Lee J. W., Newman B. A., Ferroelectric polarization mechanisms in nylon 11. Macromolecules 25, 3729–3732 (1992).

[17] Wu S. L., Scheinbeim J. I., Newman B. A., Ferroelectricity and piezoelectricity of nylon 11 films with different draw ratios. J. Polym. Sci. B Polym. Phys. 37, 2737–2746 (1999).

[18] Anwar S., Pinkal D., Zajaczkowski W., von Tiedemann P., Sharifi Dehsari H., Kumar M., Lenz T., Kemmer-Jonas U., Pisula W., Wagner M., Graf R., Frey H., Asadi K., Solution-processed transparent ferroelectric nylon thin films. Sci. Adv. 5, eaav3489 (2019). 31453321 10.1126/sciadv.aav3489 PMC6697430

[19] Jacobs E. W., Hicks J. C., Electric field induced morphological changes in nylon 11. Appl. Phys. Lett. 44, 402–403 (1984).

[20] Katz D., Gelfandbein V., Ferroelectric behaviour of α-nylon 11. J. Phys. D Appl. Phys. 15, L115–L117 (1982).

[21] Newman B. A., Chen P., Pae K. D., Scheinbeim J. I., Piezoelectricity in nylon 11. J. Appl. Phys. 51, 5161–5164 (1980).

[22] Wu G., Yano O., Soen T., Dielectric-and-piezoelectric-properties-of-nylon-9-and-nylon-11. Polym. J. 18, 51–61 (1986).

[23] Pepin J., Miri V., Lefebvre J.-M., New insights into the brill transition in polyamide 11 and polyamide 6. Macromolecules 49, 564–573 (2016).

[24] *Dassault Systèmes BIOVIA* (BIOVIA Materials Studio Visualizer, 2014).

[25] Wang Z.-Y., Fan H.-Q., Su K.-H., Wen Z.-Y., Structure and piezoelectric properties of poly(vinylidene fluoride) studied by density functional theory. Polymer 47, 7988–7996 (2006).

[26] Newman B. A., Sham T. P., Pae K. D., A high-pressure x-ray study of Nylon 11. J. Appl. Phys. 48, 4092–4098 (1977).

[27] Whiter R. A., Narayan V., Kar-Narayan S., A scalable nanogenerator based on self-poled piezoelectric polymer nanowires with high energy conversion efficiency. Adv. Energy Mater. 4, 1400519 (2014).

[28] Cauda V., Torre B., Falqui A., Canavese G., Stassi S., Bein T., Pizzi M., Confinement in oriented mesopores induces piezoelectric behavior of polymeric nanowires. Chem. Mater. 24, 4215–4221 (2012).

[29] Datta A., Choi Y. S., Chalmers E., Ou C., Kar-Narayan S., Piezoelectric Nylon-11 nanowire arrays grown by template wetting for vibrational energy harvesting applications. Adv. Funct. Mater. 27, 1604262 (2017).

[30] Shin K., Woo E., Jeong Y. G., Kim C., Huh J., Kim K.-W., Crystalline structures, melting, and crystallization of linear polyethylene in cylindrical nanopores. Macromolecules 40, 6617–6623 (2007).

[31] Michell R. M., Blaszczyk-Lezak I., Mijangos C., Müller A. J., Confinement effects on polymer crystallization: From droplets to alumina nanopores. Polym. 54, 4059–4077 (2013).

[32] Skrovanek D. J., Painter P. C., Coleman M. M., Hydrogen bonding in polymers. 2. Infrared temperature studies of nylon 11. Macromolecules 19, 699–705 (1986).

[33] Isoda H., Furukawa Y., Electric-field-induced dynamics of polymer chains in a ferroelectric melt-quenched cold-drawn film of nylon-11 using infrared spectroscopy. J. Phys. Chem. B 119, 14309–14314 (2015). 26457654 10.1021/acs.jpcb.5b08104

[34] Hu Z., Baralia G., Bayot V., Gohy J. F., Jonas A. M., Nanoscale control of polymer crystallization by nanoimprint lithography. Nano Lett. 5, 1738–1743 (2005). 16159216 10.1021/nl051097w

[35] Cauda V., Stassi S., Bejtka K., Canavese G., Nanoconfinement: An effective way to enhance PVDF piezoelectric properties. ACS Appl. Mater. Interfaces 5, 6430–6437 (2013). 23777739 10.1021/am4016878

[36] García-Gutiérrez M. C., Linares A., Hernández J. J., Rueda D. R., Ezquerra T. A., Poza P., Davies R. J., Confinement-induced one-dimensional ferroelectric polymer array. Nano Lett. 10, 1472–1476 (2010). 20232812 10.1021/nl100429u

[37] R. A. Young, *The Rietveld Method* (Oxford Univ. Press, 1995).

[38] Rietveld H. M., A profile refinement method for nuclear and magnetic structures. J. Appl. Cryst. 2, 65–71 (1969).

[39] Toraya H., Marumo F., Preferred orientation correction in powder pattern-fitting. Mineralogical J. 10, 211–221 (1981).

[40] Mei B. Z., Scheinbeim J. I., Newman B. A., The ferroelectric behavior of odd-numbered nylons. Ferroelectrics 144, 51–60 (1993).

[41] Yu H. H., Fina L. J., Electric field-induced dipole reorientation in oriented nylon 11 by in situ infrared spectroscopy. J. Polym. Sci. B Polym. Phys. 34, 781–788 (1996).

[42] Wu H., Higaki Y., Takahara A., Molecular self-assembly of one-dimensional polymer nanostructures in nanopores of anodic alumina oxide templates. Prog. Polym. Sci. 77, 95–117 (2018).

[43] Zhou Y. S., Liu Y., Zhu G., Lin Z. H., Pan C., Jing Q., Wang Z. L., In situ quantitative study of nanoscale triboelectrification and patterning. Nano Lett. 13, 2771–2776 (2013). 23627668 10.1021/nl401006x

[44] Zhou Y. S., Wang S., Yang Y., Zhu G., Niu S., Lin Z. H., Liu Y., Wang Z. L., Manipulating nanoscale contact electrification by an applied electric field. Nano Lett. 14, 1567–1572 (2014). 24479730 10.1021/nl404819w

[45] Steinhart M., Göring F., Dernaika H., Prabhukaran M., Gösele U., Hempel E., Thurn-Albrecht T., Coherent kinetic control over crystal orientation in macroscopic ensembles of polymer nanorods and nanotubes. Phys. Rev. Lett. 97, 027801 (2006). 16907479 10.1103/PhysRevLett.97.027801

[46] Ma Y., Hu W., Hobbs J., Reiter G., Understanding crystal orientation in quasi-one-dimensional polymer systems. Soft Matter 4, 540–543 (2008). 10.1039/b715065b 32907217

[47] Choi Y. S., Kim S. K., Williams F., Calahorra Y., Elliott J. A., Kar-Narayan S., The effect of crystal structure on the electromechanical properties of piezoelectric nylon-11 nanowires. Chem. Commun. 54, 6863–6866 (2018). 10.1039/c8cc02530d PMC6009497 29855641

[48] B. D. Cullity, S. R. Stock, *Elements of X-ray Diffraction* (Prentice-Hall, ed. 3, 2001).

[49] Zhang Q., Mo Z., Liu S., Zhang H., Influence of annealing on structure of nylon 11. Macromolecules 33, 5999–6005 (2000).

[50] Dasgupta S., Hammond W. B., Goddard W. A., Crystal structures and properties of nylon polymers from theory. J. Am. Chem. Soc. 118, 12291–12301 (1996).

[51] Kim K. G., Newman B. A., Scheinbeim J. I., Temperature dependence of the crystal structures of nylon 11. J. Polym. Sci. Polym. Phys. Ed. 23, 2477–2482 (1985).

[52] Kawaguchi A., Tokimitsu I., Fujiwara Y., Tabuchi M., Monobe K., Polymorphism in lamellar single crystals of nylon 11. J. Macromol. Sci. Part B Phys. 20, 1–20 (1981).

[53] Zhang Q., Mo Z., Zhang H., Liu S., Cheng S. Z. D., Crystal transitions of nylon 11 under drawing and annealing. Polymer 42, 5543–5547 (2001).

[54] Scheinbeim J. I., Piezoelectricity in γ-form nylon 11. J. Appl. Phys. 52, 5939–5942 (1981).

[55] Zhang Z., Litt M. H., Zhu L., Unified understanding of ferroelectricity in *n*-Nylons: Is the polar crystalline structure a prerequisite? Macromolecules 49, 3070–3082 (2016).

[56] Aelion R., Preparation and structure of some new types of polyamides. Ann. Chim. Appl. 3, 5–61 (1948).

[57] Nair S. S., Ramesh C., Tashiro K., Crystalline phases in nylon-11: Studies using HTWAXS and HTFTIR. Macromolecules 39, 2841–2848 (2006).

[58] Balizer E., Fedderly J., Haught D., Dickens B., Dereggi A. S., FTlR and X-ray study of polymorphs of nylon11 and relation to ferroelectricity. J. Polym. Sci. B Polym. Phys. 32, 365–369 (1994).

[59] Rhee S., White J. L., Crystalline structure and morphology of biaxially oriented polyamide-11 films. J. Polym. Sci. B Polym. Phys. 40, 2624–2640 (2002).

[60] Little K., Investigation of nylon ``texture” by X-ray diffraction. Br. J. Appl. Phys. 10, 225–230 (1959).

[61] Roguet E., Tencé-Girault S., Castagnet S., Grandidier J. C., Hochstetter G., Micromechanisms involved in the atypical tensile behavior observed in polyamide 11 at high temperature. J. Polym. Sci. B Polym. Phys. 45, 3046–3059 (2007).

[62] Slichter W. P., Crystal structures in polyamides made from ω-amino acids. J. Polym. Sci. 36, 259–266 (1959).

[63] Bunn C. W., Garner E. V., Bragg W. L., The crystal structures of two polyamides (`nylons’). Proc. R. Soc. Lond. A 189, 39–68 (1947).

[64] Holmes D. R., Bunn C. W., Smith D. J., The crystal structure of polycaproamide : Nylon 6. J. Polym. Sci. 17, 159–177 (1955).

[65] Dosiere M., Point J. J., Orientation of the boundary faces in nylon-11 lamellar crystals. J. Polym. Sci. Polym. Phys. Ed. 22, 1383–1398 (1984).

[66] Sun H., COMPASS: An ab initio force-field optimized for condensed-phase applications–Overview with details on alkane and benzene compounds. J. Phys. Chem. B 102, 7338–7364 (1998).

[67] Rappé A. K., Casewit C. J., Colwell K. S., Goddard W. A. III, Skiff W. M., UFF, a full periodic table force field for molecular mechanics and molecular dynamics simulations. J. Am. Chem. Soc. 114, 10024–10035 (1992).

[68] Jackson C. L., McKenna G. B., The melting behavior of organic materials confined in porous solids. J. Chem. Phys. 93, 9002 (1990).

[69] Lai S. L., Guo J. Y., Petrova V. V., Ramanath G., Allen L. H., Size-dependent melting properties of small tin particles: Nanocalorimetric measurements. Phys. Rev. Lett. 77, 99–102 (1996). 10061781 10.1103/PhysRevLett.77.99

[70] J. D. Menczel, L. Judovits, R. Bruce Prime, H. E. Bair, M. Reading, S. Swier, Differential Scanning Calorimetry (DSC), in *Thermal Analysis of Polymers* (Wiley-Blackwell, 2008) pp. 7–239.

[71] Lutkenhaus J. L., McEnnis K., Serghei A., Russell T. P., Confinement effects on crystallization and curie transitions of poly(vinylidene fluoride-*co*-trifluoroethylene). Macromolecules 43, 3844–3850 (2010).

[72] Jakeš J., Krimm S., A valence force field for the amide group. Spectrochim. Acta A Mol. Spectrosc. 27, 19–34 (1971).

[73] Lee G., Lee W., Lee H., Woo Lee S., Sung Yoon D., Eom K., Kwon T., Mapping the surface charge distribution of amyloid fibril. Appl. Phys. Lett. 101, 043703 (2012).

[74] Niu S., Wang S., Lin L., Liu Y., Zhou Y. S., Hu Y., Wang Z. L., Theoretical study of contact-mode triboelectric nanogenerators as an effective power source. Energ. Environ. Sci. 6, 3576–3583 (2013).

