# Supplementary material for: Unprecedented dipole alignment in α-phase nylon-11 nanowires for high-performance energy-harvesting applications
Source: Sci Adv. 2020 Jun 10;6(24):eaay5065. doi: 10.1126/sciadv.aay5065 (PMC7286685; doi:10.1126/sciadv.aay5065)
Supplement: aay5065_SM.pdf [file aay5065_SM.pdf]

Science Advances
AAAS

advances.sciencemag.org/cgi/content/full/6/24/eaay5065/DC1

Supplementary Materials for

Unprecedented dipole alignment in α-phase nylon-11 nanowires for high-performance energy-harvesting applications

Yeon Sik Choi, Sung Kyun Kim, Michael Smith, Findlay Williams, Mary E. Vickers, James A. Elliott, Sohini Kar-Narayan*

*Corresponding author. Email: sk568@cam.ac.uk

Published 10 June 2020, *Sci. Adv.* **6**, eaay5065 (2020)
DOI: 10.1126/sciadv.aay5065

This PDF file includes:

Notes S1 to S12
Figs. S1 to S19
Tables S1 to S3
References

Supplementary Note 1. Crystallography of Nylon-11

The crystal structures observed for Nylon-11 fall into two categories: (1) α, α′ and β phases; (2) pseudo-hexagonal phase (γ, γ′, δ, δ′).[50] The triclinic α-phase has been produced by precipitation from Nylon-11 solution in *m*-cresol with decreasing temperature from 150 °C to room temperature. Thus, it is considered as the most thermodynamically stable crystal structure, consisting of stacks of well-defined hydrogen-bonded sheets. The α′-phase has been achieved by slow cooling (~ 2 °C/min) of melted Nylon-11.[51] It shows almost identical crystal structures to the α-phase; a polar, triclinic unit cell with hydrogen-bonded sheets shifting progressively along the chain axis. The α′-phase can be assumed to be a defective α-form because the calculated crystal perfection index value of α′ is lower than that of α-phase.[24] In addition, the reversible phase transition at high temperature, as known as the "Brill transition", is observed only in the α′-phase, indicating that the α′-phase is a thermally tunable (*i.e.* unstable) crystal structure.[24] The β-phase was not found to be distinct, probably involving a small modification of the α phase. There is no definitive crystal structure, and this form is not of practical interest. It is generally accepted that the molecular chains in the γ-form are slightly contracted from that of the fully extended planar zigzag found in the α-form of Nylon-11. Kawaguchi et al. found that the β angle in the pseudo-hexagonal γ-phase was 118.5° (rather than 120° for perfect hexagonal crystals).[52] The δ- and δ′-phases, like γ, have a pseudo-hexagonal crystal structure. The δ-phase can be viewed as the high temperature α-form, as they are the result of the Brill transition in α′ at ~ 100 °C (quoted by Newman[26] as 95 °C, although the exact temperature is dependent on nature of the sample and heating rate).[24] It is not yet clear how the hydrogen bonding arrays are arranged in the δ-phase; the sheet-like structure from the α′-phase may or may not be preserved above the Brill transition temperature. The δ′-phase has been produced by ice quenching of melted Nylon-11, resulting in a metastable smectic pseudo-hexagonal crystal structure with a random distribution of hydrogen bonds along the chain axis. (This metastable crystal structure remains intact even when left for

extended periods of time.[53]) The relationship between δ- and δ′-phase has never been investigated, but δ′-phase is presumed to be a less ordered δ-phase because both phases show similar diffraction patterns.

Regarding ferroelectric (and piezoelectric) properties, although all the phases of Nylon-11 have a polar crystal structure due to its molecular configuration, only pseudo-hexagonal δ′-phase has been investigated extensively. This is because sparse and randomly oriented crystal structures with less hydrogen bonding in pseudo-hexagonal phase (γ, γ', δ, δ') enabled field-induced dipole reversal.[16,54,55] Whereas, in the case of α-phase, the field-induced dipole rotation was restricted up to the electrical breakdown due to the highly-packed hydrogen bonding and well-ordered crystal structure.

Supplementary Note 2. Molecular structure simulations

The family of Nylons can crystallize into several different (but similar) crystalline structures or polymorphs. The precise structure and the level of crystalline perfection depends on both the chemistry and the method of sample preparation.[24,26,50–53,56–62] The amount of crystalline perfection can be measured and ranked using several different models: the ratio of crystalline to amorphous material (usually expressed as a percentage crystallinity), crystallite size, short-range disorder and long-range disorder (or paracrystallinity). The lack of crystalline perfection shown in the classic work by Bunn was used as a starting point when modelling nylons to compare calculated data to experimental measurements.[63,64] He published atomic coordinates obtained from about 25 reflections, using data on similar nylons and chemical considerations.

The detailed molecular structure of Nylon-11 has also never been explored before due to the lack of diffraction peaks implying limited crystalline perfection and a high proportion of amorphous material. Thus, we established the most likely molecular structure using the Materials Studio 8.0 software package.[23] To investigate the molecular level configuration of Nylon-11, we created refined crystal structures of both the α-phase and the δ'-phase. First, the approximate atomic configuration of Nylon-11 was predicted based on the crystallographic information of other similar types of Nylons, such as α-Nylon-6, γ-Nylon-77, α-Nylon-66 and α-Nylon-6,10. Next, a classical and non-reactive force field-based energy optimization technique allowed us to find the most appropriate molecular geometry and bonding within the proposed unit cell parameters of Nylon-11. We then compared the calculated powder diffraction pattern of optimized molecular structure to the experimental data in the literature.

The α-phase Nylon-11 was established based on the α-phase Nylon-6,10 study by Bunn and Garner.[63] There are atomic coordinates published for this phase with parallel chains, but not a complete set for Bunn's β-phase, which has antiparallel chains but is otherwise similar to his α-phase. The initial α-Nylon-11 chain was extrapolated from an α-Nylon-6,10 model, maintaining a

tetrahedral bond angle of 109° between carbon atoms and a torsional angle of 180°. Regarding the chain conformation, we built the structure with fully extended anti-parallel molecular chains, which is the configuration suggested in the majority of Nylon-11 studies[26,59–62,65] and thus similar to Bunn's β-phase. To determine the unit cell dimensions and the indices of reflections for the triclinic α-phase Nylon-11, we compared the proposed unit cells in previous studies and took the parameters from the literature by Dosiere *et al.*[65] These unit cell parameters were then adjusted to achieve optimum matching with experimental data, as shown in Table S1.[24] With a satisfactory unit cell in place, the chain configuration was optimised using the Forcite molecular dynamics module. In the case of the α-phase with well-defined hydrogen bonded sheets, the Condensed-phase Optimized Molecular Potentials for Atomistic Simulation Studies (COMPASS)[66] force field was used to perform geometry optimisation which included the effect of electrostatic interactions modelled by partial atomic charges assigned by the force field.

The approach taken to model the smectic pseudo-hexagonal δ'-phase Nylon-11 model was different from that of the α-phase, as literature on its structure is less common.[57] The initial unit cell of δ'-Nylon-11 was created using the atomic coordination of hexagonal γ-phase Nylon-77. The *a* and *b* parameters were kept the same for the Nylon-11 model and the *c* parameter was scaled according to the change in chain length between the -77 and -11 configurations. A geometry optimisation was then conducted on the chain within the constraints of the derived unit cell. In the case of the δ'-phase, it was appropriate to use the Universal Force Field (UFF) [67] rather than COMPASS; this is because it is known that the direction of hydrogen bonding in δ'-phase is random along the chain,[24] so the simulation was set up to intentionally ignore electrostatic effects.

Supplementary Note 3. Thermally-assisted nano-template infiltration (TANI) method

Since the α-phase is the most thermodynamically stable phase of Nylon-11, typically α-phase Nylon-11 film was fabricated by method with slow crystallization speed. To achieve the 'nanowire' structured α-phase Nylon-11 with well-ordered molecular configuration, at least three different parameters, including solution employing system (related to evaporation speed), solvent vapour annealing temperature and concentration of polymer solution, should be considered and controlled simultaneously.

First of all, the crystallization of Nylon solution should be in the 'closed' system with minimized leakage of solvent vapour to reduce the solvent evaporation speed. We have placed two different glass 'lids' on the top of AAO template and of the whole system. As a result, we can limit the exposure of the solution's surface to the dry air as well as fill the environment of the system by solvent vapour, respectively. Secondly, to achieve the ordered molecular orientation using nanoconfinement effect, the polymer chain should have enough mobility. By increasing the system temperature from RT to 40 °C, we can achieve the diffraction patterns corresponding to α-phase Nylon-11 film. In addition, the intensity of α-phase peaks further increased with increasing temperature due to temperature-induced crystal ordering. This result is in a good agreement with the experimental results of Nylon-11 film, indicating that the temperature is key to changing the crystal structure of Nylon-11 nanowires. Lastly, we further diminished the crystallization speed by diluting the Nylon-11 solution. Considering the distance between polymer chains in solvent environment, diluted solution allows us to have enough time to obtain well-ordered molecular configuration in nano-pores.

It must be noted that this TANI method is different with conventional solvent vapour annealing method. To compare the both experiment, we have carried out solvent vapour annealing tests on α-phase film and δ'-phase nanowires. The test was carried out by leaving the samples in solvent (formic acid) vapour condition at room temperature or at 60 °C. Fig. S1 displays the XRD

patterns of α-phase film and δ'-phase nanowires before and after solvent vapour annealing test. The diffraction pattern of both α-phase film and δ'-phase nanowires did not change significantly even after conducting the solvent vapour annealing process at 80 °C, corresponding to the results of the thermal annealing process in the literature.[24] In that publication, Pepin et al. have been investigated the phase transition of Nylon-11 films by means of high temperature X-ray experiments. They reported that α-phase film did not show any phase transition or crystal growth after thermal annealing at 165 °C because of strong hydrogen bonding and resulting stable molecular structure. In terms of δ'-phase film, after 165 °C thermal annealing, it showed phase transition to α' during the cooling process at 75°C. These results indicate that solvent vapour annealing process does not provide enough energy to do the molecular reorganization, including crystal growth and/or phase transition. In general, the purpose of conventional solvent annealing process is additional crystal growth or phase transition of already crystallized film. However, it must be noted that TANI is the method to utilize the solvent vapour annealing method during the crystal growth to reduce the speed of crystallization, and this is the reason why we cannot see the similar phenomenon from conventional and widely-used solvent annealing process.

Supplementary Note 4. Scalability of TANI – possible schemes

1) Scale-up the size of the AAO template

In the current manuscript, α-phase nanowires fabricated by TANI method shows the peak output power of 1.062 mW, corresponding to the output power density of 3.38 $W\cdot m^{-2}$. Given the size (3.14E-4 m^2) and shape (circular shape with 1 cm diameter) of the device, 100 times enhancement of output is possible by increasing the diameter of the nanotemplate from 1 cm to 10 cm. In terms of the fabrication method, such enhancement is feasible because aforementioned capillary infiltration enables the fabrication of nanowires in large-area.

2) Scale-up using AAO fragment

Without increasing the size of AAO template, we can also scale-up this energy harvesting device by putting the fragments of AAO together. Since developed α-phase nanowires are *within* AAO template, we can easily move and assemble the nanowire-filled template pieces to produce the scale-up device. Fig. S4 shows the simple concept schematic.

3) Enhancement of power density

Two aforementioned methods are not the way to enhance the power density ($W\cdot m^{-2}$) but the way to increase the size of the device to improve the power (W). However, the power density can also be boosted by increasing the nanopores' occupied area in AAO template. We have used the AAO template with the nanopore size of 200 nm, and around 50 % of the surface area is occupied by nanopores (Fig. 2A). Considering that the nanowires grown thorough these nanopores are the performed materials for triboelectric generator, it is obvious that increasing the nanopores' occupied area can further improve the output power density of the device.

Supplementary Note 5. Crystallinity calculation

1) Calorimetry method

DSC analysis was carried out to determine the thermal and structural properties of α-phase Nylon-11 nanowires, from which the melting temperature (T_m) and the melt crystallisation temperature (T_c) were recorded. To investigate the nanoconfinement effect of nanowires, as-received α-phase films were also prepared. As shown in Fig. S5, a single melting peak at 190 °C was observed in the film (black) and nanowires without AAO template (red) during the heating cycle, in good agreement with reported values.[24]

Using the melting peaks, the crystallinity (χ) was calculated by means of the equation

$$\chi\ (\%) = \frac{\Delta H_m}{\Delta H_m^0} \times 100\ (\%)$$

where ΔH_m and $\Delta H_m^0$are the equilibrium heat of fusion enthalpies of the Nylon-11 samples and the perfect crystalline α'-phase Nylon-11 (ΔH_m^0 ~ 189 J/g)[49], respectively. ΔH_m is achieved from the area under the DSC melting peak, and ΔH_m^0 was calculated by the approach of extrapolating the linear relationship between the ΔH_m and the specific volume of samples with different degrees of crystallinity to the 100 % crystalline condition.[49] The calculated χ of both α-phase Nylon-11 films and nanowires are given in Table S2 with T_m and T_c. Although the α-phase film and nanowires give roughly the same χ of 48%, this is higher than that of α-phase film in the literature (41%).[24]

During the cooling cycle, the *Tc* of α-phase film is found at 165 °C, while nanowires displayed the *Tc* at 167 °C. The difference of the *Tc* between film and nanowires possibly arises due to the residual ordering in the polymer melts. In other words, local ordering in the melted nanowires making it easier to crystallise.

2) XRD method

The χ was also corroborated from XRD data as follows:[61]

$$\chi\,(\%) = \frac{\sum I_c}{\sum I_c + \sum I_a} \times 100\ (\%)$$

where I_c and I_a are the integrated intensities scattered over a suitable angular interval by the crystalline and the non-crystalline (*i.e.* amorphous) region, respectively. As shown in Fig. 2D, XRD patterns of α-phase Nylon-11 film and nanowires without AAO template show one amorphous peak at ~ 21.4° and three distinct crystalline peaks at 2θ of 7.8°, 20°, and 24.2°. The relative intensities and area of all the crystalline and amorphous peaks were calculated based on profile fitting results. Both the film and nanowires show almost identical crystallinities with an average χ of ~ 37 ± 2 %, a trend similar to that obtained by calorimetry method.

Supplementary Note 6. Thermal behaviour of Nylon-11 nanowires

To prove the nanoconfinement effect, we have explored the thermal and structural properties of the series of Nylon-11, including α-phase film, δ′-phase films fabricated by melt-press or drop-casting method, nanowires fabricated by conventional template wetting method, α-phase nanowires fabricated by TANI method and δ′-phase nanowires fabricated by gas-assited nano-template infiltration method. All nanowire samples were prepared with and without AAO template. As shown in Fig. S6, during the heating cycle, a single melting peak at 190 °C was observed in the α-phase (orange) and melt-quenched δ′-phase films (grey), in a good agreement with reported values.[24] (Here, we would like to skip the detailed explanation about the solution-based δ′-phase films.) In the case of nanowires *within* AAO, the α-phase showed the melting peak at 189 °C that is 1 °C lower than that of films. The δ′-phase nanowire also displayed decreased melting peaks at 185 °C and 189 °C, and the difference between the two melting peaks became more distinct. The T_m depression found in the nanowires in the AAO template would be explained by the Gibbs-Thomson equation:[68,69]

$$\Delta T_m = T_m - T_m(d) = \frac{4\,\sigma_{sl}\,T_m}{d\,\Delta H_f\,\rho_s}$$

where ΔT_m is the melting point depression, T_m the normal (bulk) melting point, $T_m(d)$ the melting point of cylindrical nanocrystals with diameter d, σ_{sl} the surface tension of the solid-liquid interface, ΔH_f the bulk enthalpy of fusion (per g of material), and ρ_s the concentration of the solid. This equation is well-matched with the T_m suppression of 1 ~ 2 °C observed in the nanowires relative to films because ΔT_m is inversely proportional to d. When the nanowires are freed from the AAO template, the melting behaviour of α-phase nanowires transited to bimodal melting, maintaining the dominant T_m at 189 °C. Furthermore, position of the main melting peak of δ′-phase nanowires is altered from 185 °C to 189 °C. The melting behaviour of nanowires

fabricated by conventional template wetting method was also manipulated with a similar trend to α-phase nanowires. According to the literature, several sources can be responsible for multiple melting peaks:[70] 1) recrystallisation during melting; 2) multiple crystalline phases exist simultaneously; 3) phase transition occurring during heating; and 4) removal of defects or improvement of ordering in the crystal structures by annealing effect. In the case of Nylon-11 nanowires without AAO, it is possible to experience recrystallisation during the melting process. This is because when nanowires are in the template, the thermal behaviour is restricted by the template wall, the crystal perfections then possibly changes during template dissolving process. Interestingly, the T_m of nanowires (*within* the AAO) fabricated by conventional template wetting method was achieved at 191 °C that is much higher than that of α- and δ′-nanowires. Furthermore, the crystallinity of this un-reported phase of Nylon-11 is 40 %, which is much higher than that of δ′-phase nanowires (38 %), meaning that nanowires fabricated by conventional template wetting also have stable and highly crystalline phase.

During the cooling cycle, films and template-freed nanowires with the same phases exhibited similar crystallisation behaviour (Fig. S7). The T_c of α-phase film is found near 165 °C, and template freed α-phase nanowires displayed a T_c at around 166 °C. The solution-based δ′-phase film and nanowires displayed a T_c at around 164 °C. The difference of the T_c between α- and δ′-phase possibly arise due to the residual ordering in the polymer melts. In contrast, *within* the template, the Nylon-11 showed different crystallisation behaviour with relatively weak and broad crystallisation peak at around 156 °C. This is because homogeneous nucleation occurred in the nano-sized pores. As discussed by Lutkenhaus et al., heterogeneous nucleation is typified by a sharp crystallisation peak upon cooling in DSC, which we observe here for Nylon-11 films and template-freed nanowires.[71] A broad and supercooled crystallisation transition are attributed to the homogeneous nucleation where there are a few defects to initiate crystal growth. It must be noted that, in the case of PVDF or P(VDF-TrFE), such homogenous nucleation was only observed when

they were confined in very small size of pores (< 50 nm).[71] (For bulk or crystallisation in bigger pores (~ 200 nm), P(VDF-TrFE) presented the sharp crystallisation peak corresponding to heterogeneous nucleation.) This is because the size of nanostructures should be smaller than the natural average distance between heterogeneous nucleation centres to achieve the complete confinement.[34] In contrast, in the case of Nylon-11, homogeneous nucleation is observed in 200 nm pores, indicating that the distance between nucleation centres of Nylon-11 is much bigger than that of PVDF and its copolymers. Above all, this means that the complete nanoconfinement of Nylon-11 can be achieved via 200 nm AAO template.

Supplementary Note 7. FT-IR measurement

The FT-IR spectra of α-phase nanowires and film are presented in Fig. S8. The peak positions and their assignments are listed in Table S3.[32,33,72] The α-phase nanowires show the same peak intensity at 2850 cm^{-1} and much higher intensities for the peaks at 3300, 2920, 1635, and 1537 cm^{-1} than those of film. The band at 3300 cm^{-1} is assigned to N-H stretching vibration and is sensitive to the strength of the hydrogen bond,[32] and the relative peak intensities between 2850 and 3300 cm^{-1} in film and α-phase nanowires are 0.60 and 0.69, respectively. Considering that the deviations of relative peak intensities within the same spectrum show the changes in chemical bonds, this difference indicates that more hydrogen bonds are inside the nanowires than film. The IR region around 1640 ~ 1680 cm^{-1} is assigned to the amide I mode, which is conformationally related to dipole-dipole interactions.[32] As a result, deconvolution of the amide I band, especially band at 1635 cm^{-1}, can provide information regarding ordered and disordered hydrogen-bonded conformation. Given that the relative peak intensities between 2850 and 1635 cm^{-1} in film and α-phase nanowires are 2.18 and 2.01, respectively, it can be inferred that the hydrogen bonding in α-phase nanowires resembles much more an ordered crystal structure than that in the film.

Supplementary Note 8. A rocking curve on the (200) reflection

To confirm the preferential orientation of polymer chains, XRD rocking curves (or omega (ω) scans) were measured. While keeping 2θ constant, the scattering vector (q) is ‘rocked’ or rotated by scanning ω. For an unoriented sample, the intensity should remain constant as shown by the background scan (Fig. S9, blue) which only has a fall off at ends as first the incident and then the exit angle approach zero. (The background data was taken at 2θ = 22° where is away from the (200) peak.) However, the (200) reflection (2θ = 20°) exhibits a real maximum when its scattering vector q is aligned along the axis of the nanowires (Fig. S9, red), indicating that crystallites are orientated such that the scattering vectors tend to be perpendicular to the sample surface. This result confirm that preferred orientation is the cause of the loss of intensity of the other peaks and increase in (200).

Supplementary Note 9. KPFM measurement

The KPFM measurements were carried out in the 'noncontact mode' with a 2 V alternating current (AC) signal. Fig. S10. shows the basic operational mechanism of KPFM.[73] On the first trace, the topography of the sample is recorded by the mechanical vibration of the cantilever. The cantilever is then lifted to the 'lift scan height (z_h)' and performed the second scan whilst maintaining a constant separation (z_h) of the cantilever from the measured topology, thus compensating the contribution from topography. (The z_h can be controlled manually and change the measured values of surface potential. We set the lift scan height ranging from 20 to 50 nm.) During the second scan, the mechanical vibration is turned off and an oscillating voltage ($= V_{AC} \sin \omega t$) is applied directly to the tip, where V_{AC} is the AC voltage amplitude, ω the resonant frequency of the cantilever, and t the time. If we assume that a conductive cantilever tip and a conductive sample form a capacitor structure, the energy (U) between tip and sample is derived by parallel plate capacitor model:

$$U = \frac{1}{2} C (\Delta V)^2$$

where C the local capacitance between the AFM tip and the sample, ΔV the voltage difference between the two. Since the ΔV consists of direct current (DC) and oscillator induced AC component, ΔV can be described as follows:

$$\Delta V = \Delta V_{DC} + V_{AC} \sin \omega t$$

where ΔV_{DC} includes applied DC voltage (V_{DC}) from the feedback loop, work function differences (V_{CPD}), surface charge effects, etc (V_{etc}). The electrostatic force (F_{el}) between tip and the sample can then be expressed as a function of the separation distance (z):

$$F_{el} = \frac{1}{2}\frac{\partial C}{\partial z}(\Delta V)^2$$

This means that the local surface potential can be achieved by varying the V_{DC} applied to the tip. Between tip and sample, if ΔV is non-zero, the cantilever experiences F_{el}, and amplitude can be detected during vibration. However, if ΔV become zero by adjusting V_{DC}, cantilever amplitude will be zero. As a result, the relatively weak but long-range electrostatic interactions can be measured, minimizing the influence of topography.

Supplementary Note 10. Device performance of triboelectric energy generator

Fig. S16A shows the electrical power output of the α-phase nanowire-based triboelectric generators across different resistors. The electrical output was found to increase with increasing impact amplitude and frequencies as expected (Fig. S16B, C). Fatigue testing was carried out by continuously impacting the device for up to 30 hours at 5 Hz (≈ 540,000 cycles), showing that the α-phase Nylon-11 nanowire-based triboelectric generator exhibited negligible change in output current density over the entire period of continuous testing (Fig. S16D).

Supplementary Note 11. Triboelectric charge transfer process

In the dielectric-to-dielectric contact-separation mode triboelectric generators (Fig. S18), the electric field strength (*E*) of one dielectric part is derived by Gauss's law and given by

$$E = \frac{Q}{S\,\varepsilon_0\,\varepsilon_r}$$

where Q is the value of the transferred charges between the two electrodes, S the area of the electrode, ε_0 the permittivity of the vacuum (8.854×10^{-12} F m^{-1}), and ε_r the relative permittivity (*i.e.* dielectric constant) of dielectric material.[74] The electric potential difference (ΔV) between two electrodes can be thus given by

$$\begin{aligned}\Delta V &= E_1\,d_1 + E_2\,d_2 + E_{air}\,x(t)\\ &= \left[-\frac{Q}{S\,\varepsilon_0\,\varepsilon_{r1}}\,d_1\right] + \left[-\frac{Q}{S\,\varepsilon_0\,\varepsilon_{r2}}\,d_2\right] + \left[\frac{S\,\sigma - Q}{S\,\varepsilon_0}\,x(t)\right]\\ &= -\frac{Q}{S\,\varepsilon_0}\left(\frac{d_1}{\varepsilon_{r1}} + \frac{d_2}{\varepsilon_{r2}} + x(t)\right) + \frac{\sigma\,x(t)}{\varepsilon_0}\end{aligned}$$

where d_1 and d_2 are the thickness of the two dielectric materials, $x(t)$ the distance between two contact surfaces, t the time, σ the transferred charge density (*i.e.* triboelectric charge density), and ε_{r1} and ε_{r2} the dielectric constants of the two dielectric materials. (In the conductor-to-dielectric structure, t_1/ε_{r1} can be ignored because the metal layer acts as both triboelectric layer and electrode.) Under open-circuit conditions, the value of transferred charge (Q) becomes zero since no charge is transferred between electrodes. Thus, if we assume the electric potential of the bottom electrode to be zero, the equation for the open-circuit voltage (V_{oc}) can be calculated to be

$$V_{oc} = \frac{\sigma}{\varepsilon_0}\, x(t)$$

From the experimentally determined V_{oc} of each triboelectric energy harvester, the theoretical transferred charge density (σ) can be estimated by

$$\sigma = \frac{V_{oc}\, \varepsilon_0}{x(t)}$$

It must be noted that the amount of transferred charge density does not indicate all charge on the surface of materials. The amount of transferred charge was determined by charge affinity, and a number of factors, such as electron affinity, electronegativity, surface roughness, influence on charge affinity (*i.e.* triboelectricity) simultaneously. The influence of spontaneous polarization on the performance of the triboelectric generator has been previously studied.[7–9] According to these studies, positively poled PVDF and its copolymer films showed more than double the power output when incorporated into triboelectric generators as compared to the un-poled film. This is due to an increase in surface charge density as a result of the spontaneous polarization. In other words, more screening charge (*i.e.* unbound charge) are attached on the surface of the materials with spontaneous polarization to generate the equilibrium states, and such increasing of screening charge also increase the amount of transferred charge during contact and separation motion. Therefore, removal of the charge from the surface does not lead the depolarization of Nylon-11 nanowires.

Supplementary Note 12. The piezoelectricity of α-phase Nylon-11 nanowires

First of all, as we have demonstrated in the manuscript, it is obvious that the developed α-phase nanowires have preferential crystal orientation. The XRD results well-corresponded to the trends in other publications regarding polymer nanoconfinement, and we also proved such crystal orientation with additional evidence, including matching with molecular simulation results and rocking curve-based analysis. Secondly, as the calculated crystallinity of the nanowires (48 %) supported, this α-phase Nylon-11 nanowire is a 'semi-crystalline' material. It must be noted that having preferential crystal orientation in nanowires does not indicate that developed α-phase nanowires have to be a 'single crystal' material. Thirdly, from the piezoelectric force microscopy (PFM) analysis, we have not seen the piezoelectric response from this α-phase nanowires. In our previous publication,[47] the mechanical and electric properties of these nanowires were extensively explored using quantitative nanomechanical mapping (QNM) and PFM, and the α-phase nanowires showed the highest Young's modulus but did not exhibit significant piezoelectric behaviour. In contrast, a distinct piezoelectric response was achieved from the relatively soft δ'-phase nanowires, and a similar trend in the variation of properties across the different crystal phases were also observed in Nylon-11 films. This is the reason why we did define this material as a 'well-ordered α-phase Nylon-11 nanowires' instead of 'piezoelectric material'. Lastly, without piezoelectric properties, the developed α-phase nanowires could be used to improve the performance of triboelectric energy generators because of its dipole alignment. As we stated above, recent investigations have proposed that spontaneous polarization can enhance the output performance of triboelectric devices.[7–9] Furthermore, even in the case of P(VDF-TrFE), which is definite piezoelectric (and ferroelectric) material, the output performance of triboelectric energy generator is ~ 20 times higher than that of the piezoelectric generator when the experiment was conducted using the same materials with the same input forces. It indicates that the contribution of triboelectricity is more dominant and higher than that of piezoelectricity. Besides, to maximise

the piezoelectric device performance, metal/polymer/metal capacitor structure has to be used, whereas, in our manuscript, gold electrode was deposited only one side of the nanowire-based devices (Fig. S19), which is the standard configuration for triboelectric generator, and this energy harvesting setup would further diminish the contribution of piezoelectricity from whole output performance as the piezoelectric capacitor structure is avoided. For these reasons, we can conclude that the contribution of piezoelectricity is negligible and have defined α-phase nanowires-based device as a ‘triboelectric energy generator’.

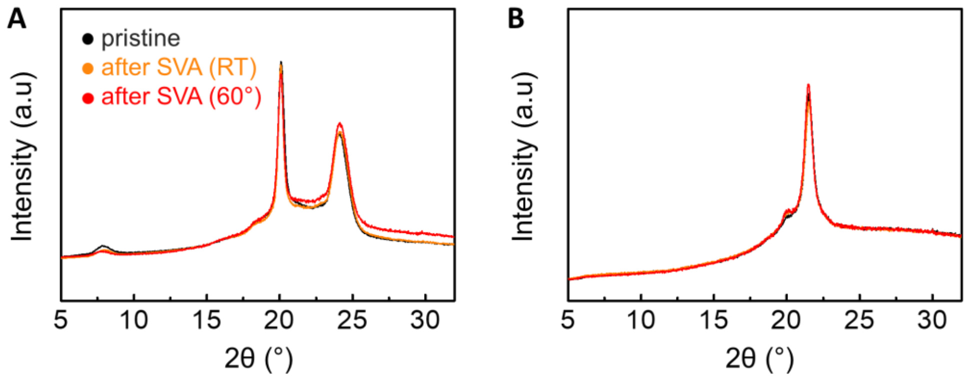

Fig. S1. Changes of XRD patterns of Nylon-11 nanowires before and after solvent vapour annealing process. Comparison of XRD patterns of (black) pristine sample, (orange) sample after solvent vapour annealing (SVA) at room temperature (RT) and after SVA at 60 °C. (A) α-phase Nylon-11 film and (B) δ′-phase Nylon-11 nanowires *within* AAO template.

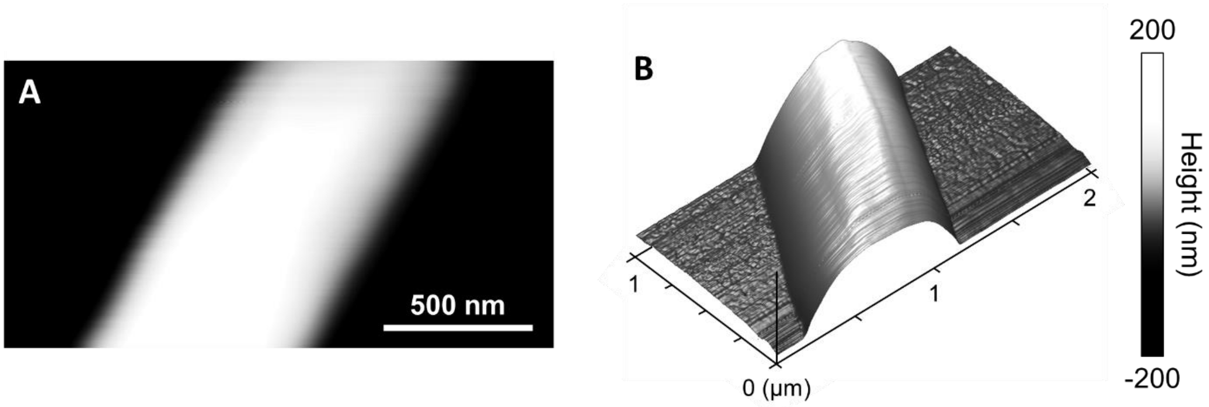

Fig. S2. Topography of Nylon-11 nanowire strand. (A) 2D and **(B)** 3D AFM topography image of a single nanowire strand fabricated by TANI method. This shows that a highly dense and smooth surface (without grain boundaries) can be obtained by the TANI method.

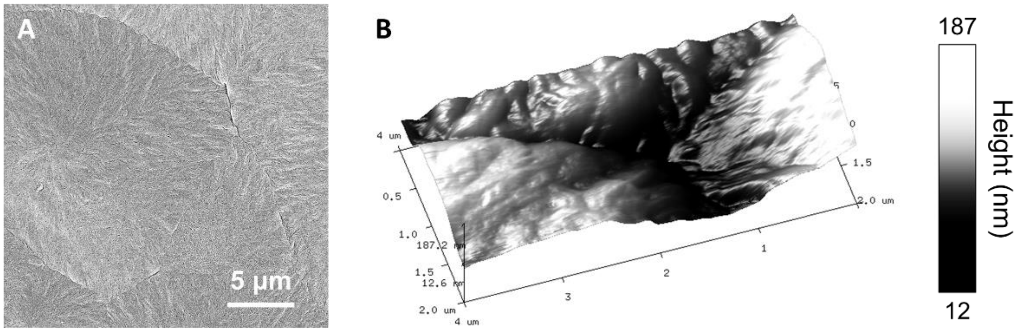

Fig. S3. Surface morphology of α-phase Nylon-11 film. (A) SEM image displaying the top surface of the film. **(B)** AFM topography image of a continuous thin film.

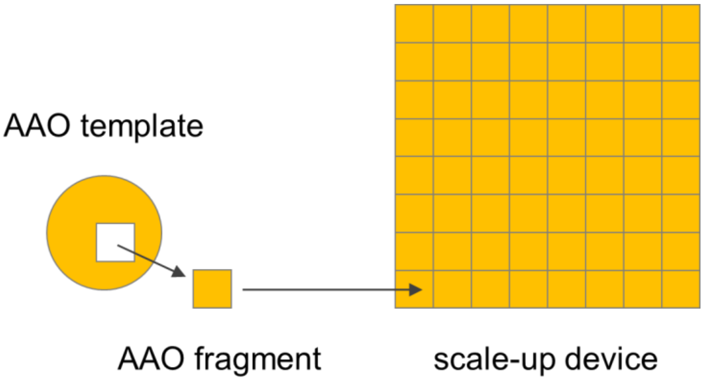

Fig. S4. Schematics of the scale-up procedure by using AAO fragment.

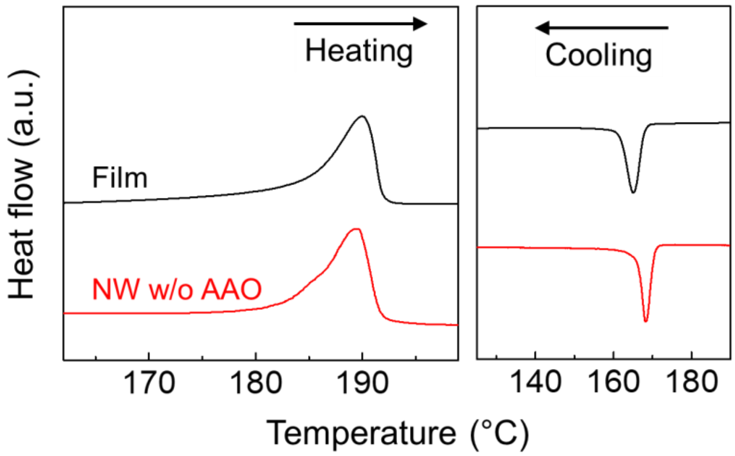

Fig. S5. DSC thermogram for (black) the α-phase film and (red) the template-freed α-phase nanowires.

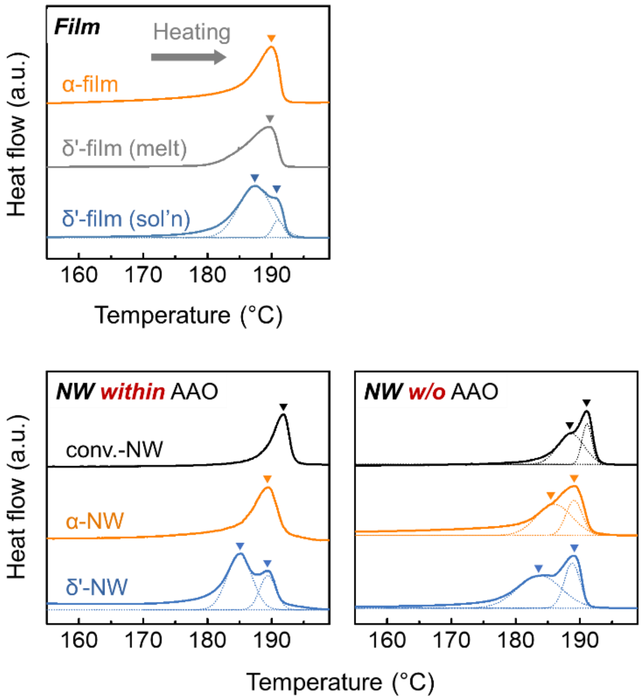

Fig. S6. DSC thermograms obtained during the first heating. Four different crystal structures of films and nanowires were investigated: (orange) α-, (grey) melt-quenched δ′-, and (blue) solution-based δ′-phase films and nanowires. (black) un-reported phase from nanowires fabricated by conventional template-wetting.

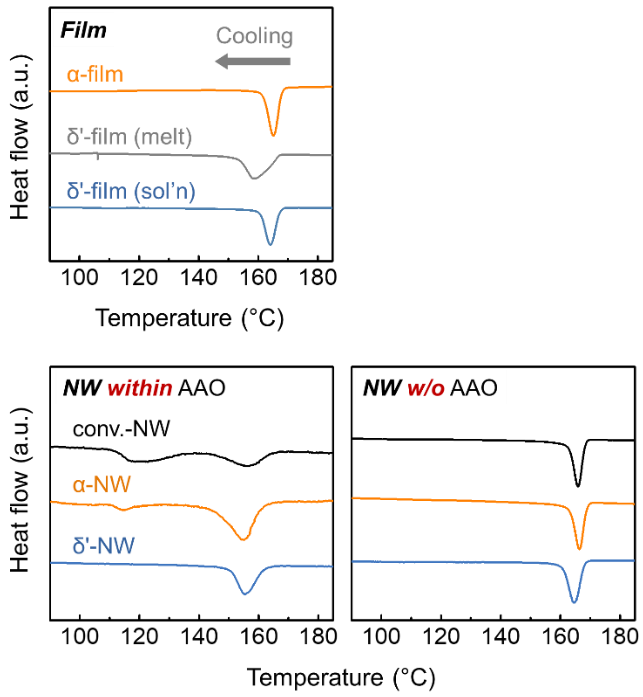

Fig. S7. DSC thermograms obtained during the cooling. Four different crystal structures of films and nanowires were investigated: (orange) α-, (grey) melt-quenched δ′-, and (blue) solution-based δ′-phase films and nanowires. (black) un-reported phase from nanowires fabricated by conventional template-wetting.

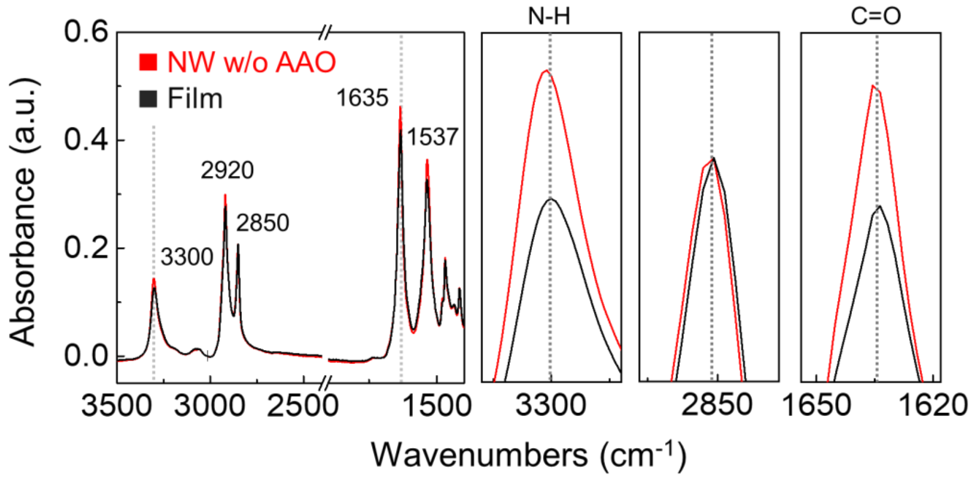

Fig. S8. FT-IR absorbance. IR spectra of α-phase (red) template-freed nanowires and (black) film. Right graphs indicate the infrared spectra near N-H stretching (3300 cm^{-1}), CH_2 symmetric stretching (2850 cm^{-1}) and amide I (1635 cm^{-1}) modes, respectively.

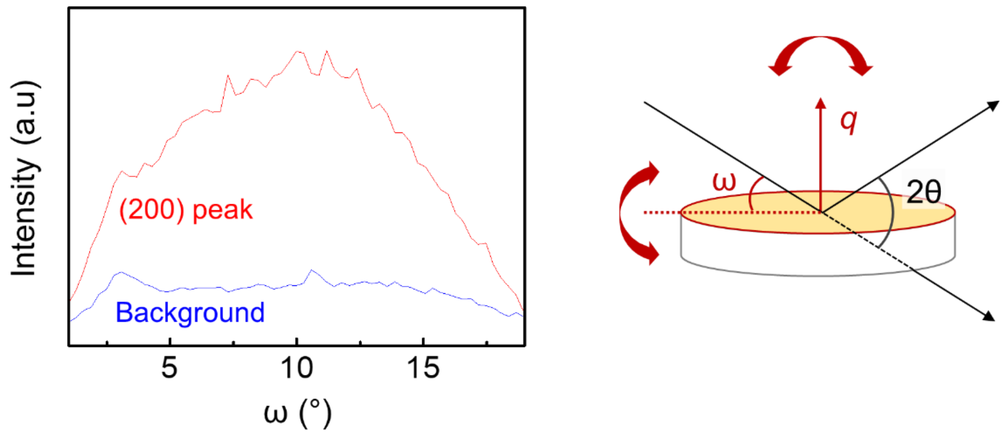

Fig. S9. XRD rocking curve. A rocking curve performed on the (red) (200) peak and (blue) background observed from nanowires within a template.

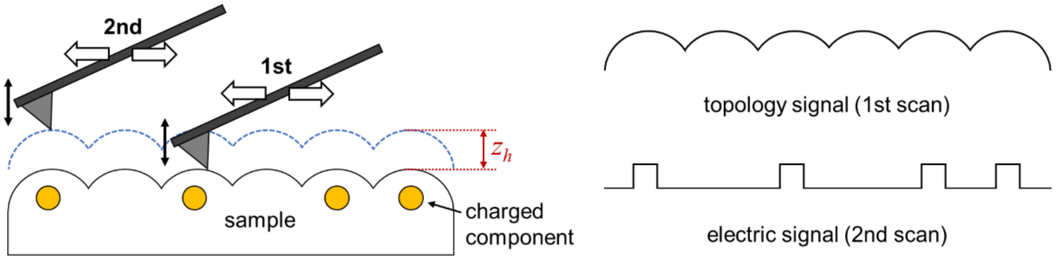

Fig. S10. Schematics for the operational mechanisms of KPFM. (left) First topology scan in tapping mode and 2nd interleave scan with the lift scan height (z_h). (right) Resulting topology and electrical signals during the 1st and 2nd scanning process.

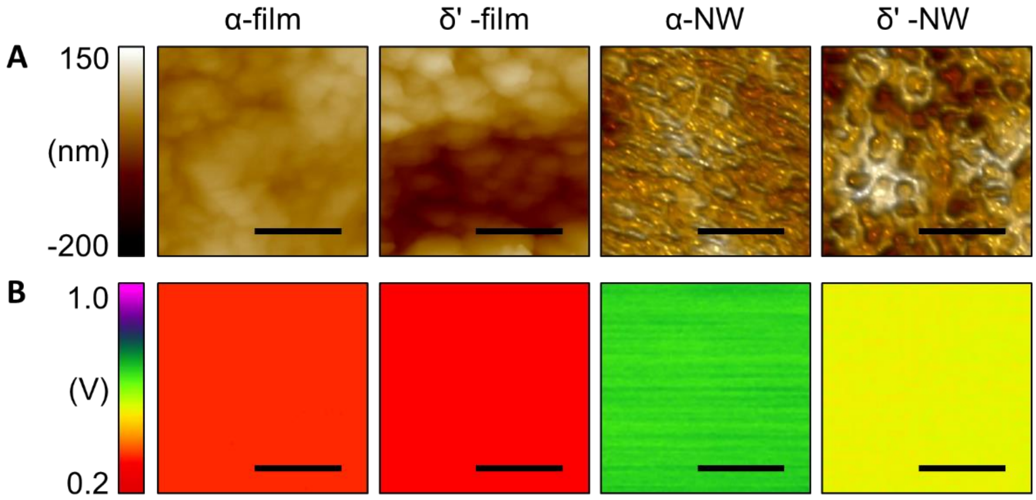

Fig. S11. KPFM analysis for Nylon-11 films and nanowires. (A) AFM topology images of Nylon-11 films and nanowires. **(B)** Surface potential of each samples: α-film (20 mV), δ′-film (184 mV), α-NW (576 mV), and δ′-NW (395 mV). Scale bar indicates 1 μm.

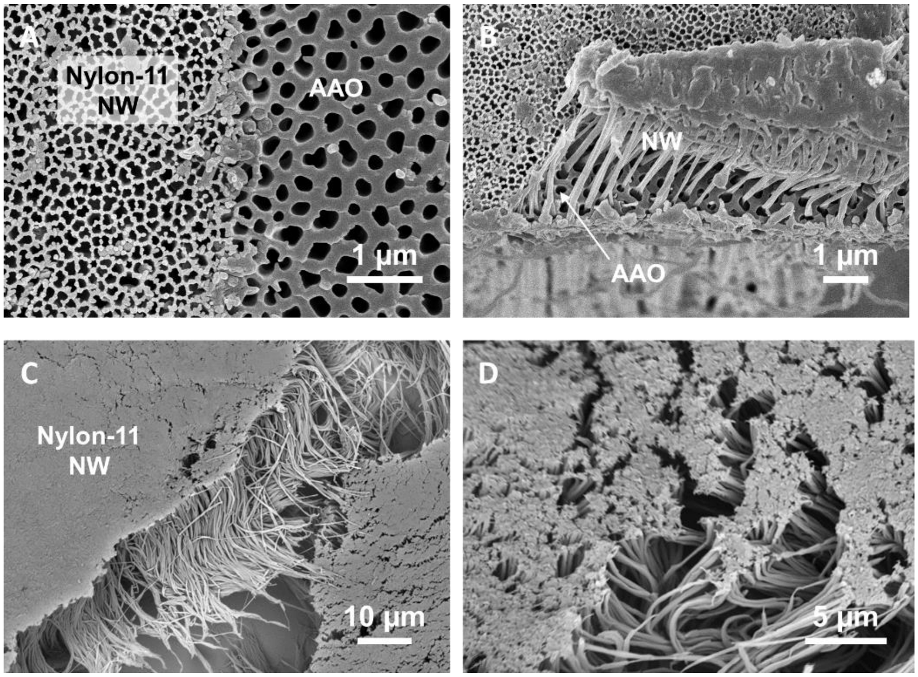

Fig. S12. SEM images of Nylon-11 nanowires (A,B) with and (C,D) without AAO template.

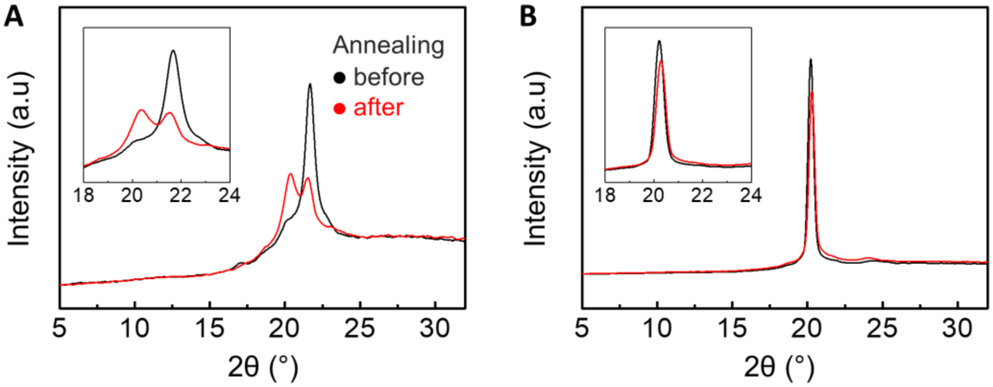

Fig. S13. Thermal stability of Nylon-11 nanowires – Comparison of XRD patterns (black) before and (red) after thermal annealing at 165 °C. **(A)** δ′-phase Nylon-11 nanowires *within* AAO template. **(B)** α-phase Nylon-11 nanowires *within* AAO template. Inset displays the magnified area from 2θ of 18° to 2θ of 24°.

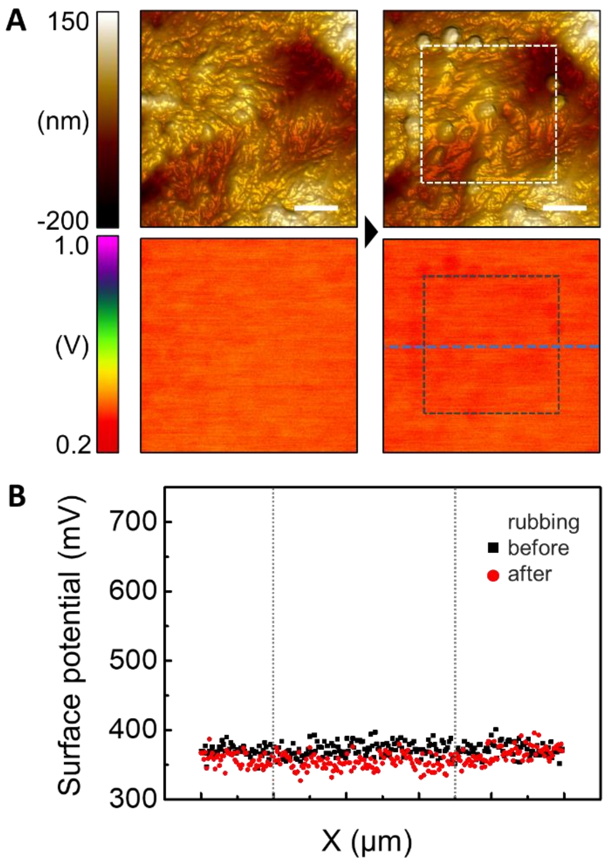

Fig. S14. **Surface potential analysis of α-phase film before and after rubbing process. (A)** (above) AFM topology and (below) surface potential images (left) before and (right) after rubbing process. Dotted squares indicate rubbing area. Scale bar indicates 1 μm. Blue dot line indicates the data acquisition position for plotting. **(B)** Surface potential changes (black square) before and (red circle) after rubbing process.

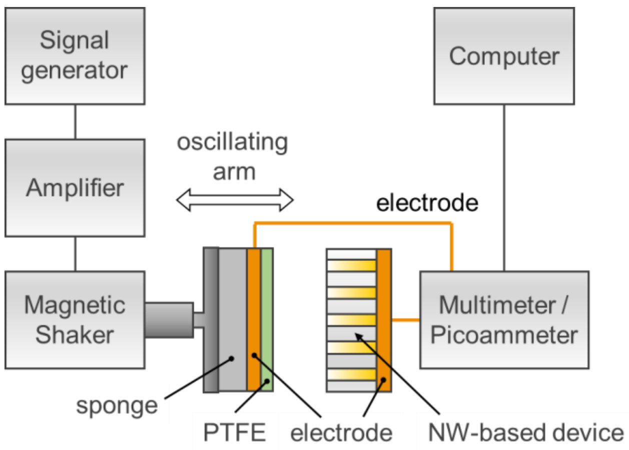

Fig. S15. Schematics of triboelectric energy harvesting setup.

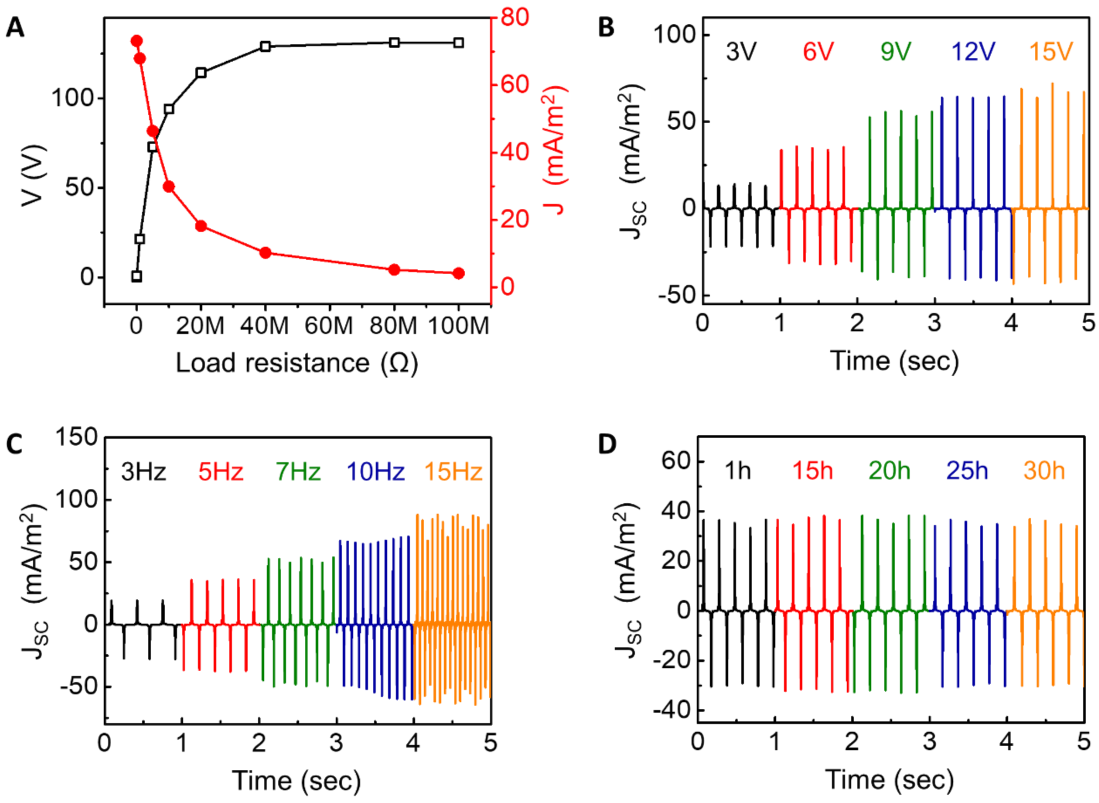

Fig. S16. Triboelectric energy generator device performance. (A) The change in (black) voltage and (red) current density of α-phase nanowires-based triboelectric generators across the various load resistors. **Various input condition. (B, C)** The short circuit current density of the α-phase nanowire device was measured under the application of a periodic impacting force between

3 ~ 15 V with constant frequency of 5 Hz and a variable frequency between 3 ~ 15 Hz with constant amplitude of 6 V. In our energy generator system, the force amplitude of the magnetic shaker could be controlled via the applied voltage. **Fatigue test. (D)** Short circuit current density recorded over time in response to continuous impacting at a frequency of 5 Hz and amplitude of 6 V on the same α-phase nanowire-based triboelectric generator device for 30 h (~ 540,000 cycles impacting cycles in total). Data were recorded after 1 h (18 k cycles), 15 h (270 k cycles), 20 h (360 k cycles), 25 h (450 k cycles), and 30 h (540 k cycles).

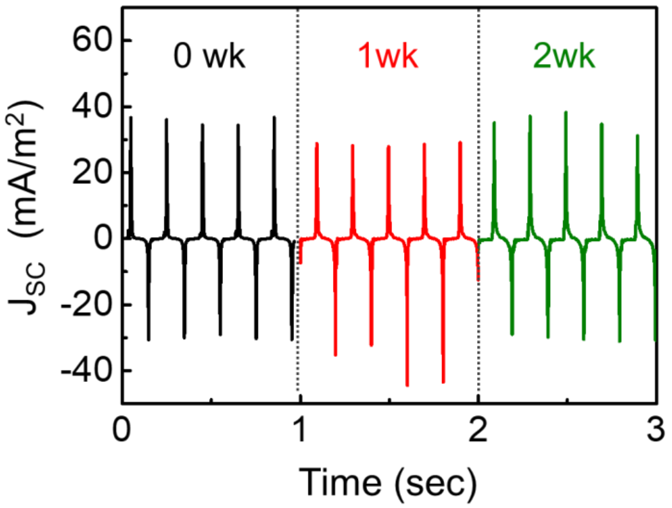

Fig. S17. Long-term reliability test. Short circuit current density recorded over time in response to continuous impacting at a frequency of 5 Hz and amplitude of 6 V on the same α-phase nanowire-based triboelectric generator device. The output performance was measured after resting the device in the lab environment for 1 and 2 weeks.

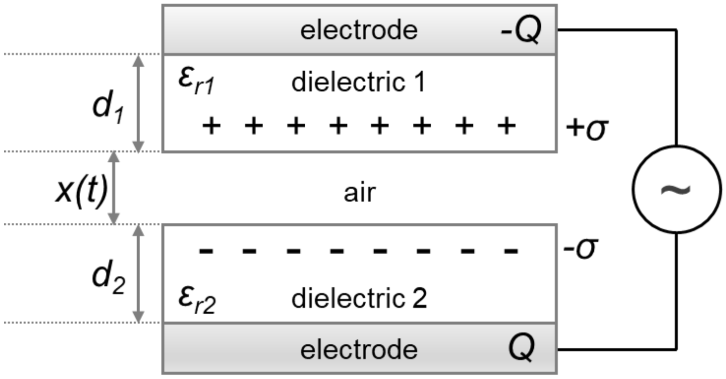

Fig. S18. Schematic of the vertical contact-separation mode triboelectric energy generator.

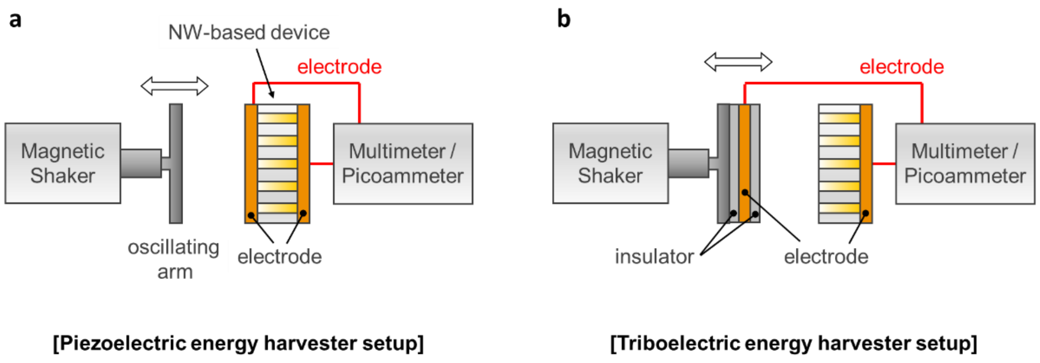

Fig. S19. Schematics of both piezoelectric and triboelectric energy harvesting setup; (a) piezoelectric and (b) triboelectric energy harvesting devices.

Table S1. Nylon-11 unit cell parameters. A comparison between the unit cell parameters determined by Dosiere *et al.*, and the altered set used to match the simulated XRD pattern with the experimental data.

	**a (Å)**	**b (Å)**	**c (Å)**	**α (°)**	**β (°)**	**γ (°)**
**Reference (α)**[65]	9.65	5.25	14.9	50.5	90.0	72.0
**Model fit to experimental data (α)**	9.75	5.15	15.1	51.5	90.0	71.0

Table S2. DSC results. Melting point (T_m), melt crystallisation temperature (T_c), and degree of crystallinity (χ) of α-phase Nylon-11. (Accuracy ± 1 °C and 2 %)

	T_m (°C)	T_c (°C)	χ (%)
**Film**	190	165	48
**NW**	190	167	48

Table S3. FT-IR absorbance list of Nylon-11. Region, assignments and peak positions (cm^{-1}) of the infrared bands of Nylon-11

**Region**	**assignments**	**peak (cm^{-1})**
amide A	NH stretching	3309
	overtone of amide I	3275
	combination of amide I and amide II	3197
CH_2 stretching	antisymmetric stretching	2923
	symmetric stretching	2850
amide I	non-H bonded (free)	1676
	H-bonded (disordered / amorphous)	1646
	H-bonded (ordered / crystalline)	1638
	unassigned	1623
amide II	H-bonded	1558
	H-bonded	1540
	free	1516
CH_2 scissoring	NH-vicinal CH_2 scissoring	1477
	CH_2 scissoring (in-phase)	1469
	CH_2 scissoring	1457
	CH_2 scissoring	1437
	CO-vicinal CH_2 scissoring	1419
amide III	amide III	1374
	CH_2 rocking	721

REFERENCES AND NOTES

1. A. J. Lovinger, Ferroelectric polymers. *Science* **220**, 1115–1121 (1983).

2. H. S. Nalwa, *Ferroelectric Polymers: Chemistry: Physics, and Applications* (Marcel Dekker Inc., 1995).

3. S. Horiuchi, Y. Tokura, Organic ferroelectrics. *Nat. Mater.* **7**, 357–366 (2008).

4. L. Persano, C. Dagdeviren, Y. Su, Y. Zhang, S. Girardo, D. Pisignano, Y. Huang, J. A. Rogers, High performance piezoelectric devices based on aligned arrays of nanofibers of poly(vinylidenefluoride-co-trifluoroethylene). *Nat. Commun.* **4**, 1633 (2013).

5. A. H. Rajabi, M. Jaffe, T. L. Arinzeh, Piezoelectric materials for tissue regeneration: A review. *Acta Biomater.* **24**, 12–23 (2015).

6. R. F.Valentini, T. G. Vargo, J. A. Gardella Jr., P. Aebischera, Electrically charged polymeric substrates enhance nerve fibre outgrowth in vitro. *Biomaterials* **13**, 183–190 (1992).

7. J.-H. Lee, R. Hinchet, T. Y. Kim, H. Ryu, W. Seung, H. J. Yoon, S. W. Kim, Control of skin potential by triboelectrification with ferroelectric polymers. *Adv. Mater.* **27**, 5553–5558 (2015).

8. K. Y. Lee, S. K. Kim, J.-H. Lee, D. Seol, M. K. Gupta, Y. Kim, S.-W. Kim, Controllable charge transfer by ferroelectric polarization mediated triboelectricity. *Adv. Funct. Mater.* **26**, 3067–3073 (2016).

9. W. Seung, H.-J. Yoon, T. Y. Kim, H. Ryu, J. Kim, J.-H. Lee, J. H. Lee, S. Kim, Y. K. Park, Y. J. Park, S.-W. Kim, Boosting power-generating performance of triboelectric nanogenerators via artificial control of ferroelectric polarization and dielectric properties. *Adv. Energy Mater.* **7**, 1600988 (2016).

10. J. Wang, C. Wu, Y. Dai, Z. Zhao, A. Wang, T. Zhang, Z. L. Wang, Achieving ultrahigh triboelectric charge density for efficient energy harvesting. *Nat. Commun.* **8**, 88 (2017).

11. Y. S.Choi, Q. Jing, A. Datta, C. Boughey, S. Kar-Narayan, A triboelectric generator based on self-poled Nylon-11 nanowires fabricated by gas-flow assisted template wetting. *Energ. Environ. Sci.* **10**, 2180–2189 (2017).

12. M. Li, H. J. Wondergem, M. J. Spijkman, K. Asadi, I. Katsouras, P. W. Blom, D. M. de Leeuw, Revisiting the δ-phase of poly(vinylidene fluoride) for solution-processed ferroelectric thin films. *Nat. Mater.* **12**, 433–438 (2013).

13. J. W. Lee, Y. Takase, B. A. Newman, J. I. Scheinbeim, Effect of annealing on the ferroelectric behavior of nylon-11 and nylon-7. *J. Polym. Sci. B Polym. Phys.* **29**, 279–286 (1991).

14. Y. Takahashi, M. Shimomura, M. Kutani, T. Furukawa, Ferroelectric switching characteristics and crystal structure of nylon 11. *Polym. J.* **29**, 234–239 (1997).

15. J. W. Lee, Y. Takase, B. A. Newman, J. I. Scheinbeim, Ferroelectric polarization switching in nylon-11. *J. Polym. Sci. B Polym. Phys.* **29**, 273–277 (1991).

16. J. I. Scheinbeim, J. W. Lee, B. A. Newman, Ferroelectric polarization mechanisms in nylon 11. *Macromolecules* **25**, 3729–3732 (1992).

17. S. L. Wu, J. I. Scheinbeim, B. A. Newman, Ferroelectricity and piezoelectricity of nylon 11 films with different draw ratios. *J. Polym. Sci. B Polym. Phys.* **37**, 2737–2746 (1999).

18. S. Anwar, D. Pinkal, W. Zajaczkowski, P. von Tiedemann, H. Sharifi Dehsari, M. Kumar, T. Lenz, U. Kemmer-Jonas, W. Pisula, M. Wagner, R. Graf, H. Frey, K. Asadi, Solution-processed transparent ferroelectric nylon thin films. *Sci. Adv.* **5**, eaav3489 (2019).

19. E. W. Jacobs, J. C. Hicks, Electric field induced morphological changes in nylon 11. *Appl. Phys. Lett.* **44**, 402–403 (1984).

20. D. Katz, V. Gelfandbein, Ferroelectric behaviour of α-nylon 11. *J. Phys. D Appl. Phys.* **15**, L115–117 (1982).

21. B. A. Newman, P. Chen, K. D. Pae, J. I. Scheinbeim, Piezoelectricity in nylon 11. *J. Appl. Phys.* **51**, 5161–5164 (1980).

22. G. Wu, O. Yano, T. Soen, Dielectric-and-piezoelectric-properties-of-nylon-9-and-nylon-11. *Polym. J.* **18**, 51–61 (1986).

23. J. Pepin, V. Miri, J.-M. Lefebvre, New insights into the brill transition in polyamide 11 and polyamide 6. *Macromolecules* **49**, 564–573 (2016).

24. *Dassault Systèmes BIOVIA* (BIOVIA Materials Studio Visualizer, 2014).

25. Z.-Y. Wang, H.-Q. Fan, K.-H. Su, Z.-Y. Wen, Structure and piezoelectric properties of poly(vinylidene fluoride) studied by density functional theory. *Polymer* **47**, 7988–7996 (2006).

26. B. A. Newman, T. P. Sham, K. D. Pae, A high-pressure x-ray study of Nylon 11. *J. Appl. Phys.* **48**, 4092–4098 (1977).

27. R. A. Whiter, V. Narayan, S. Kar-Narayan, A scalable nanogenerator based on self-poled piezoelectric polymer nanowires with high energy conversion efficiency. *Adv. Energy Mater.* **4**, 1400519 (2014).

28. V. Cauda, B. Torre, A. Falqui, G. Canavese, S. Stassi, T. Bein, M. Pizzi, Confinement in oriented mesopores induces piezoelectric behavior of polymeric nanowires. *Chem. Mater.* **24**, 4215–4221 (2012).

29. A. Datta, Y. S. Choi, E. Chalmers, C. Ou, S. Kar-Narayan, Piezoelectric Nylon-11 nanowire arrays grown by template wetting for vibrational energy harvesting applications. *Adv. Funct. Mater.* **27**, 1604262 (2017).

30. K. Shin, E. Woo, Y. G. Jeong, C. Kim, J. Huh, K.-W. Kim, Crystalline structures, melting, and crystallization of linear polyethylene in cylindrical nanopores. *Macromolecules* **40**, 6617–6623 (2007).

31. R. M. Michell, I. Blaszczyk-Lezak, C. Mijangos, A. J. Müller, Confinement effects on polymer crystallization: From droplets to alumina nanopores. *Polym.* **54**, 4059–4077 (2013).

32. D. J. Skrovanek, P. C. Painter, M. M. Coleman, Hydrogen bonding in polymers. 2. Infrared temperature studies of nylon 11. *Macromolecules* **19**, 699–705 (1986).

33. H. Isoda, Y. Furukawa, Electric-field-induced dynamics of polymer chains in a ferroelectric melt-quenched cold-drawn film of nylon-11 using infrared spectroscopy. *J. Phys. Chem. B* **119**, 14309–14314 (2015).

34. Z. Hu, G. Baralia, V. Bayot, J. F. Gohy, A. M. Jonas, Nanoscale control of polymer crystallization by nanoimprint lithography. *Nano Lett.* **5**, 1738–1743 (2005).

35. V. Cauda, S. Stassi, K. Bejtka, G. Canavese, Nanoconfinement: An effective way to enhance PVDF piezoelectric properties. *ACS Appl. Mater. Interfaces* **5**, 6430–6437 (2013).

36. M. C. García-Gutiérrez, A. Linares, J. J. Hernández, D. R. Rueda, T. A. Ezquerra, P. Poza, R. J. Davies, Confinement-induced one-dimensional ferroelectric polymer array. *Nano Lett.* **10**, 1472–1476 (2010).

37. R. A. Young, *The Rietveld Method* (Oxford University Press, 1995).

38. H. M. Rietveld, A profile refinement method for nuclear and magnetic structures. *J. Appl. Cryst.* **2**, 65–71 (1969).

39. H. Toraya, F. Marumo, Preferred orientation correction in powder pattern-fitting. *Mineralogical J.* **10**, 211–221 (1981).

40. B. Z. Mei, J. I. Scheinbeim, B. A. Newman, The ferroelectric behavior of odd-numbered nylons. *Ferroelectrics* **144**, 51–60 (1993).

41. H. H. Yu, L. J. Fina, Electric field-induced dipole reorientation in oriented nylon 11 by in situ infrared spectroscopy. *J. Polym. Sci. B Polym. Phys.* **34**, 781–788 (1996).

42. H. Wu, Y. Higaki, A. Takahara, Molecular self-assembly of one-dimensional polymer nanostructures in nanopores of anodic alumina oxide templates. *Prog. Polym. Sci.* **77**, 95–117 (2018).

43. Y. S. Zhou, Y. Liu, G. Zhu, Z. H. Lin, C. Pan, Q. Jing, Z. L. Wang, In situ quantitative study of nanoscale triboelectrification and patterning. *Nano Lett.* **13**, 2771–2776 (2013).

44. Y. S. Zhou, S. Wang, Y. Yang, G. Zhu, S. Niu, Z. H. Lin, Y. Liu, Z. L. Wang, Manipulating nanoscale contact electrification by an applied electric field. *Nano Lett.* **14**, 1567–1572 (2014).

45. M. Steinhart, F. Göring, H. Dernaika, M. Prabhukaran, U. Gösele, E. Hempel, T. Thurn-Albrecht, Coherent kinetic control over crystal orientation in macroscopic ensembles of polymer nanorods and nanotubes. *Phys. Rev. Lett.* **97**, 027801 (2006).

46. Y. Ma, W. Hu, J. Hobbs, G. Reiter, Understanding crystal orientation in quasi-one-dimensional polymer systems. *Soft Matter* **4**, 540–543 (2008).

47. Y. S. Choi, S. K. Kim, F. Williams, Y. Calahorra, J. A. Elliott, S. Kar-Narayan, The effect of crystal structure on the electromechanical properties of piezoelectric nylon-11 nanowires. *Chem. Commun.* **54**, 6863–6866 (2018).

48. B. D. Cullity, S. R. Stock, *Elements of X-ray Diffraction* (Prentice-Hall, ed. 3, 2001).

49. Q. Zhang, Z. Mo, S. Liu, H. Zhang, Influence of annealing on structure of nylon 11. *Macromolecules* **33**, 5999–6005 (2000).

50. S. Dasgupta, W. B. Hammond, W. A. Goddard, Crystal structures and properties of nylon polymers from theory. *J. Am. Chem. Soc.* **118**, 12291–12301 (1996).

51. K. G. Kim, B. A. Newman, J. I. Scheinbeim, Temperature dependence of the crystal structures of nylon 11. *J. Polym. Sci. Polym. Phys. Ed.* **23**, 2477–2482 (1985).

52. A. Kawaguchi, I. Tokimitsu, Y. Fujiwara, M. Tabuchi, K. Monobe, Polymorphism in lamellar single crystals of nylon 11. *J. Macromol. Sci. Part B Phys.* **20**, 1–20 (1981).

53. Q. Zhang, Z. Mo, H. Zhang, S. Liu, S. Z. D. Cheng, Crystal transitions of nylon 11 under drawing and annealing. *Polymer* **42**, 5543–5547 (2001).

54. J. I. Scheinbeim, Piezoelectricity in γ-form nylon 11. *J. Appl. Phys.* **52**, 5939–5942 (1981).

55. Z. Zhang, M. H. Litt, L. Zhu, Unified understanding of ferroelectricity in *n*-Nylons: Is the polar crystalline structure a prerequisite? *Macromolecules* **49**, 3070–3082 (2016).

56. R. Aelion, Preparation and structure of some new types of polyamides. *Ann. Chim. Appl.* **3**, 5–61 (1948).

57. S. S. Nair, C. Ramesh, K. Tashiro, Crystalline phases in nylon-11: Studies using HTWAXS and HTFTIR. *Macromolecules* **39**, 2841–2848 (2006).

58. E. Balizer, J. Fedderly, D. Haught, B. Dickens, A. S. Dereggi, FTlR and X-ray study of polymorphs of nylon11 and relation to ferroelectricity. *J. Polym. Sci. B Polym. Phys.* **32**, 365–369 (1994).

59. S. Rhee, J. L. White, Crystalline structure and morphology of biaxially oriented polyamide-11 films. *J. Polym. Sci. B Polym. Phys.* **40**, 2624–2640 (2002).

60. K. Little, Investigation of nylon ``texture'' by X-ray diffraction. *Br. J. Appl. Phys.* **10**, 225–230 (1959).

61. E. Roguet, S. Tencé-Girault, S. Castagnet, J. C. Grandidier, G. Hochstetter, Micromechanisms involved in the atypical tensile behavior observed in polyamide 11 at high temperature. *J. Polym. Sci. B Polym. Phys.* **45**, 3046–3059 (2007).

62. W. P. Slichter, Crystal structures in polyamides made from ω-amino acids. *J. Polym. Sci.* **36**, 259–266 (1959).

63. C. W. Bunn, E. V. Garner, W. L. Bragg, The crystal structures of two polyamides (`nylons'). *Proc. R. Soc. Lond. A* **189**, 39–68 (1947).

64. D. R. Holmes, C. W. Bunn, D. J. Smith, The crystal structure of polycaproamide : Nylon 6. *J. Polym. Sci.***17**, 159–177 (1955).

65. M. Dosiere, J. J. Point, Orientation of the boundary faces in nylon-11 lamellar crystals. *J. Polym. Sci. Polym. Phys. Ed.* **22**, 1383–1398 (1984).

66. H. Sun, COMPASS: An ab initio force-field optimized for condensed-phase applications–Overview with details on alkane and benzene compounds. *J. Phys. Chem. B* **102**, 7338–7364 (1998).

67. A. K. Rappé, C. J. Casewit, K. S. Colwell, W. A. Goddard III, W. M. Skiff, UFF, a full periodic table force field for molecular mechanics and molecular dynamics simulations. *J. Am. Chem. Soc.* **114**, 10024–10035 (1992).

68. C. L. Jackson, G. B. McKenna, The melting behavior of organic materials confined in porous solids. *J. Chem. Phys.* **93**, 9002 (1990).

69. S. L. Lai, J. Y. Guo, V. V. Petrova, G. Ramanath, L. H. Allen, Size-dependent melting properties of small tin particles: Nanocalorimetric measurements. *Phys. Rev. Lett.* **77**, 99–102 (1996).

70. J. D. Menczel, L. Judovits, R. Bruce Prime, H. E. Bair, M. Reading, S. Swier, Differential Scanning Calorimetry (DSC), in *Thermal Analysis of Polymers* (Wiley-Blackwell, 2008) pp. 7–239.

71. J. L. Lutkenhaus, K. McEnnis, A. Serghei, T. P. Russell, Confinement effects on crystallization and curie transitions of poly(vinylidene fluoride-*co*-trifluoroethylene). *Macromolecules* **43**, 3844–3850 (2010).

72. J. Jakeš, S. Krimm, A valence force field for the amide group. *Spectrochim. Acta A Mol. Spectrosc.* **27**, 19–34 (1971).

73. G. Lee, W. Lee, H. Lee, S. Woo Lee, D. Sung Yoon, K. Eom, T. Kwon, Mapping the surface charge distribution of amyloid fibril. *Appl. Phys. Lett.* **101**, 043703 (2012).

74. S. Niu, S. Wang, L. Lin, Y. Liu, Y. S. Zhou, Y. Hu, Z. L. Wang, Theoretical study of contact-mode triboelectric nanogenerators as an effective power source. *Energ. Environ. Sci.* **6**, 3576–3583 (2013).
